# Clinical Outcomes on Home Parenteral Support for Patients with Benign Chronic Intestinal Failure: Systematic Review and Meta-Analysis

**DOI:** 10.3390/nu18132123

**Published:** 2026-07-01

**Authors:** Maja Eckhardt, Sorrel Burden, Eilidh McGowan, Gordon L. Carlson, Loris Pironi, Simon Lal

**Affiliations:** 1 Intestinal Failure Unit, Northern Care Alliance NHS Foundation Trust, Salford M6 8HD, UK; sorrel.burden@manchester.ac.uk (S.B.); eilidh.mcgowan.10@gmail.com (E.M.); gordon.carlson@nca.nhs.uk (G.L.C.); simon.lal@nca.nhs.uk (S.L.); 2 School of Health Sciences, University of Manchester, Manchester M13 9PL, UK; 3 Department of Medical and Surgical Sciences, University of Bologna, 40126 Bologna, Italy; loris.pironi@unibo.it

**Keywords:** intestinal failure, home parenteral support, mortality, nutritional autonomy

## Abstract

**Background/Objectives**: Chronic intestinal failure (IF) necessitating home parenteral support (HPS) represents an end-stage condition for many gastrointestinal diseases. While long-term outcomes have been reported in HPS-dependent patients, there are limited generalisable data on survival and nutritional autonomy rates in large contemporary cohorts. The primary objective of this study was to examine the evidence on mortality and nutritional autonomy rates for HPS published to 2025. **Methods**: This is a systematic review and meta-analysis (PROSPERO Registration number: CRD42025645325). A database search was conducted up to 3 February 2025. Studies assessing survival and nutritional autonomy in adults with benign chronic IF receiving HPS were included. For clinical outcomes, crude proportions were calculated and a meta-analysis was performed to calculate the proportion affected per year of follow-up, with survival probabilities additionally presented on Kaplan–Meier curves. **Results**: Overall, 3411 records were screened and 57 studies were included. The overall mortality was 8.0% per year of follow-up (95% CI 6.0 to 10.0%), or 33.5% over a mean of 49.02 months. The summary of survival curves estimated the pooled five- and ten-year survival rates from HPS initiation as 68.71% and 52.46%, respectively. Nutritional autonomy was achieved in 35.3% of patients, at a rate of 10.0% per year of follow-up (95% CI 7.0% to 14.0%). Age, gastrointestinal anatomy, and underlying disease leading to IF were identified as potential factors associated with mortality and nutritional autonomy. **Conclusions**: This is the first systematic review and meta-analysis summarising the current literature on survival and nutritional autonomy in adults with chronic IF. These findings increase our understanding of this complex condition, providing baseline data to support clinical decision-making and patient counselling.

## 1. Introduction

Intestinal failure (IF) is a complex and debilitating condition defined as the inability of the gut to absorb sufficient macronutrients, electrolytes, and water to sustain life, such that intravenous supplementation or ‘parenteral support’ is required [1]. Before the late 1960s, a diagnosis of IF was considered incompatible with life; however, the subsequent development of intravenous nutrition has transformed IF care [2]. Intestinal failure can be classified according to functional, pathophysiological, and clinical features: type 1 is an acute, short-term, and usually self-limiting condition; type 2 is a prolonged acute condition, typically in metabolically unstable patients following surgery, which may last for weeks or months; type 3, or chronic IF, describes the need, reversible or irreversible, for long-term parenteral support in metabolically stable patients [1,3].

The pathophysiological mechanisms leading to chronic IF include short bowel syndrome (SBS), gastrointestinal dysmotility, intestinal fistulae, mechanical obstruction, and extensive mucosal disease and can result from a variety of underlying conditions, such as Crohn’s disease, mesenteric ischaemia, surgical complications, or chronic intestinal pseudo-obstruction [1]. Indeed, given its complexity, chronic IF is often considered to be the end-stage of many gastrointestinal diseases, requiring cohesive and specialised multi-disciplinary input for safe long-term care [4,5]. Thus, ideally, patients with chronic IF should be able to receive parenteral support at home (termed ‘home parenteral support or HPS’), which typically involves administering intravenous solutions via a pump over 10 to 14 h, between two and seven nights per week.

In Europe, the prevalence of chronic IF due to benign disease has been estimated to range from 5 to 80 cases per million of the national population, and it has been steadily increasing over the last four decades, due to both increased awareness and advances in the surgical management and critical care of patients [4,6]. However, there remains global inequity of access to HPS and, in many countries worldwide, no treatment options at all for patients with chronic IF [7]. Where treatment is available, it is recognised that the approach to delivery of care for patients with chronic IF differs both within and between countries, which has an impact on long-term patient outcomes [8,9]. Monitoring and reporting of the outcomes are crucial since, although essential for life, the impact of HPS on both the individual [10] and the healthcare economy is high [11]. Indeed, complications include those associated with the central venous catheter, such as catheter-related bloodstream infections or thrombosis, as well as IF-associated liver disease and metabolic bone problems [12,13,14]. Moreover, treatment with HPS has been shown to have a substantial negative impact on patients’ quality of life due to the highly regimented lifestyle required [15,16]. Overall, the cost of HPS in adult patients in Europe has been estimated as between €13,000 and €71,000 per year, including HPS bags cost, personnel cost, and/or consumables cost [11].

Historical cohort studies have reported 5-year survival probabilities of between 58% and 83% for patients on HPS [17,18,19,20]. Previous research also shows that chronic IF is associated with more than seven-fold higher mortality rates than for the general UK population and shorter life expectancies by more than 17 years [18]. Nevertheless, despite increased awareness of this condition and its complications, as well as advances in novel therapies, there are no recent comparisons between patient outcomes among centres. Indeed, the most recent narrative review describing the probability of survival and nutritional autonomy in adults with chronic IF included studies only up to 2011 [21]. In addition, the latter review was not performed systematically and did not include a meta-analysis, and pooled survival and nutritional autonomy estimates were not calculated. Importantly, since its publication, there have been developments in data interpretation that raise questions about the optimal study design for the correct derivation and interpretation of the analysis of outcomes in patients with chronic IF [22].

The primary aim of this systematic review and meta-analysis was therefore to examine the evidence published up to 2025 on mortality and nutritional autonomy in HPS-dependent patients with chronic IF due to benign disease. The secondary aim was to examine the evidence on factors associated with increasing the rate of these clinical outcomes, as well as evidence on the primary causes of death in this patient cohort.

## 2. Materials and Methods

### 2.1. Reporting Guidelines and Protocol Registration

This systematic review and meta-analysis followed the PRISMA 2020 guidance for reporting systematic reviews. The PRISMA 2020 checklist can be found in Supplementary Table S1. In accordance with the guidelines, our systematic review protocol was registered with the International Prospective Register of Systematic Reviews (PROSPERO) on 29 January 2025 (Registration number: CRD42025645325).

### 2.2. Eligibility Criteria

Population: We included observational studies including cohort and cross-sectional studies that studied adult patients with benign chronic IF receiving HPS. Clinical trials were excluded because they include highly selected samples of patients, frequently undergoing additional tests or interventions, including promoting weaning off HPS with medication, making their results less comparable to general cohorts of patients with IF. Studies with individuals all under the age of 18 were excluded. If the underlying disease leading to IF in the included subjects was benign for more than 80% of participants, or if data were able to be extracted for the subgroup of participants with benign aetiology of IF, these studies were included.

Exposures: Exposures that included the provision of HPS.

Outcomes: The outcomes included mortality and regaining nutritional autonomy. Patients were classified as having achieved nutritional autonomy if they remained off HPS for a period of 12 months or, if these data were not available, when defined by the authors as “weaned off HPS.” Additional outcomes included factors associated with mortality and nutritional autonomy, as well as primary causes of death categorised as IF-related, underlying disease-related, non-HPS/non-IF-related, or unknown. No attempt was made to associate nutritional autonomy with glucagon-like peptide 2 therapies. Outcomes were extracted as reported in all included studies and in all data forms using a pre-defined data extraction form.

Setting: Studies taking place only in home settings were included. Studies taking place in the hospital setting were excluded.

Years, Language, and Publication Status: There were no publication date restrictions. Articles reported in any language and describing original research published in peer-reviewed scientific journals were included.

### 2.3. Information Sources and Search Strategy

Searches were conducted up to 3 February 2025. The databases included MEDLINE (Ovid interface, U.S. National Library of Medicine), EMBASE (Ovid interface, Elsevier), and Web of Science (Clarivate Analytics interface, Clarivate). To ensure literature saturation, PROSPERO was searched for any ongoing or completed reviews. The reference lists from all relevant reviews were searched. Additionally, the reference and citation lists of all included studies were manually screened to identify any other relevant articles. Literature search strategies were developed using medical subject headings (MeSH) and text words related to *intestinal failure*, *home parenteral support*, *mortality*, and *nutritional autonomy*. The full search strategy can be found in Supplementary Figure S1.

### 2.4. Selection Process

Two reviewers (M.K. and E.M.) independently screened the titles and abstracts to exclude irrelevant studies. The full text was obtained for any studies that met the inclusion criteria or where there was any uncertainty. The full texts were then assessed for the inclusion/exclusion criteria. Disagreements between the reviewers were resolved through discussion and arbitration with a third author (S.L.) if necessary. We did not need to contact any study authors to clarify eligibility. The reasons for excluding studies were noted.

### 2.5. Data Collection Process

The literature search results were uploaded to Rayyan software version 1.7.5 (web application) for organisation and screening. Any duplicates were identified and removed automatically and then manually in Rayyan software. Summary estimate data were extracted from the included studies using a standardised data extraction form, developed a priori. Extracted variables included centre, country, study years, inclusion and exclusion criteria, study design, sample size, mean patient age, proportion of male patients, median and range of HPS duration, mortality rate, nutritional autonomy rate, and causes of death. Data extraction was carried out by one reviewer (M.K.) and then verified by another (E.M.) to reduce bias and errors.

### 2.6. Data Outcomes

The main outcomes of interest were mortality and nutritional autonomy. These were measured as the number of events per year of follow-up. Data on reported associations of clinical outcomes were extracted from each included study, and whether associations had been established using a univariate or multivariate analysis. The data on mechanism and underlying disease leading to IF were extracted as reported in the studies. Short bowel syndrome was frequently sub-classified in the studies based on the remaining gastrointestinal anatomy as group 1 (small bowel end-ostomy), group 2 (jejunocolic anastomosis), and group 3 (jejunoileocolic anastomosis with intact ileocecal valve and colon) [1]. Other extracted data included author, study years, country, IF centre name, study design, sample size, mean age, sex, duration of follow-up, duration of HPS treatment, and causes of death.

### 2.7. Study Risk-of-Bias Assessment

Two reviewers (M.K. and S.B.) independently assessed the risk of bias by applying the Risk of Bias in Non-Randomised Studies of Exposures (ROBINS-E) tool. The judgement was either ‘Low’ or ‘High’ risk of bias, or ‘Some Concerns’.

### 2.8. Effect Measures and Synthesis of Data

To reduce the risk of duplication of patients in the meta-analysis, studies were selected for inclusion if their reported patient outcomes did not overlap with those of other reported cohorts. The author names, centre names and locations, and years of inclusion were compared such that only non-overlapping cohorts were included in the meta-analysis.

We only conducted meta-analyses if there were studies reporting similar comparisons for the outcome measures. For clinical outcomes (mortality and nutritional autonomy), crude proportions were calculated and then meta-analysis was performed to calculate the proportion affected per year of follow-up. Random effects were used throughout, with 95% CIs. Heterogeneity was calculated according to Cochrane’s Q statistic and its related metric I^2^. Analysis was performed using the R package *meta* version 8.2-1 in R Version 4.5.2.

Additionally, a summary of survival probabilities presented on Kaplan–Meier curves was undertaken using the method described by Combescure et al. [23]. In brief, the survival probabilities were read from the survival curves from the time of the HPS initiation until the end of follow-up. For this purpose, the pictures of the curves were digitalised, and the probabilities were extracted using the package *SurvdigitizeR* version 0.0.0.9000. Then, the numbers of at-risk patients during the different time intervals were assessed from the total sample size, number-at-risk patient tables, and survival probabilities using the R package *IPDfromKM* version 0.1.10. Finally, the *MetaSurvival* version 0.1.0 method was applied over a follow-up of ten years with random effects, with 95% CIs to visualise the summarised survival curves.

## 3. Results

### 3.1. Study Selection

**Figure 1:**
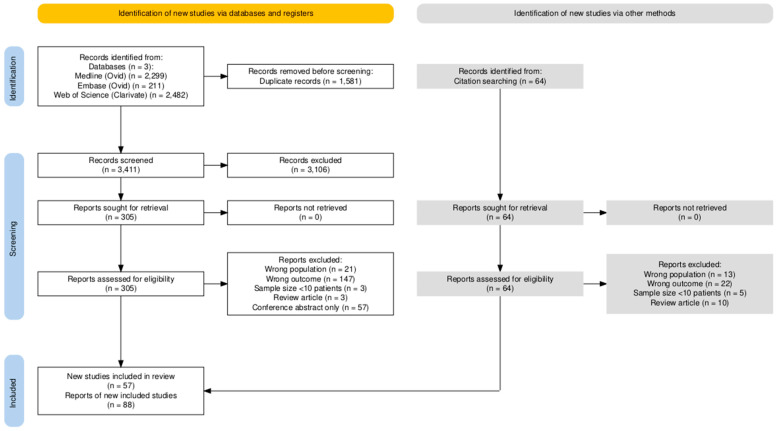
PRISMA 2020 flow diagram for a systematic review of the outcomes in patients with chronic intestinal failure receiving home parenteral support.

We found a total of 4992 records from searching the databases and, after removing duplicates, 3411 records were screened. From these, 305 full-text documents were reviewed, and 74 reports were included. Additionally, a further 64 records were identified from scanning the reference lists of initially included studies and relevant prior reviews. After checking for duplicates, we reviewed the 64 additional reports and included 14, resulting in a total of 88 included records. These represented 57 separate adult study populations [6,9,18,19,20,22,24,25,26,27,28,29,30,31,32,33,34,35,36,37,38,39,40,41,42,43,44,45,46,47,48,49,50,51,52,53,54,55,56,57,58,59,60,61,62,63,64,65,66,67,68,69,70,71,72,73,74], due to some cohorts overlapping. When multiple reports were published on one cohort, only the most recent or complete report was included. There were a further six papers that reported data concerning risk factors for mortality [75,76] and nutritional autonomy [77,78] as well as cause of death [79,80] that were not published in the primary article arising from that study, meaning that we extracted data from 63 separate articles in total. The PRISMA flow diagram, including the reasons for reports’ exclusion, can be viewed in Figure 1.

### 3.2. Study Characteristics

The characteristics of the included studies are presented in Table 1. Included studies were from Argentina (*n* = 1) [69], Australia (*n* = 1) [32], Belgium (*n* = 1) [51], Brazil (*n* = 1) [56], Canada (*n* = 1) [68], China (*n* = 1) [61], Denmark (*n* = 5) [6,22,37,46,59], France (*n* = 6) [20,24,38,39,52,55], Italy (*n* = 4) [43,47,53,72], Japan (*n* = 4) [31,35,57,66], the Netherlands (*n* = 2) [58,63], Portugal (*n* = 2) [71,73], Spain (*n* = 1) [45], the UK (*n* = 9) [18,27,30,34,44,49,54,67], the USA (*n* = 12) [25,26,29,33,40,42,48,50,64,65,70,74], Europe (*n* = 4) [28,36,41,60], and worldwide (*n* = 2) [9,62]. In terms of study design, the vast majority (50 out of 57) were retrospective cohort studies [6,9,18,19,20,22,25,26,28,29,30,31,32,33,34,36,37,38,39,40,41,42,43,44,45,46,47,48,49,51,52,53,55,56,57,58,59,60,61,62,64,65,66,67,70,71,72,73,74], six were prospective cohort studies [24,27,54,63,68,69], and one a retrospective cross-sectional study [35]. The majority of the studies (36 out of 57) included mixed populations of patients with different mechanisms leading to chronic IF [6,9,18,19,24,25,26,27,28,30,32,34,35,36,37,39,40,41,43,44,45,46,47,48,49,50,51,53,54,56,58,62,63,67,71,72], twelve studies included only patients with chronic IF due to SBS [22,31,38,42,55,59,61,65,68,69,70,73], five only patients with chronic IF due to Crohn’s disease/inflammatory bowel disease [33,52,57,64,66,74], one only patients with chronic IF due to chronic intestinal pseudo-obstruction [20], one only patients with chronic IF due to post-bariatric complications [60], and one only patients with chronic IF due to scleroderma [29].

Overall, 34 studies were single- [6,18,19,22,25,27,29,30,31,32,33,39,40,42,43,44,48,49,50,51,55,56,58,59,61,63,64,65,66,70,71,72,73,74] and 23 were multicentre [9,20,24,26,28,34,35,36,37,38,41,45,46,47,52,53,54,57,60,62,67,68,69]. Forty studies reported the mean patient age at HPS initiation, ranging from 20 to 64 years [6,9,18,19,20,22,24,28,32,33,35,38,39,40,43,44,45,46,47,48,49,50,51,54,55,57,58,59,60,61,62,63,65,66,67,69,71,72,73,74]. Twenty-four studies reported the mean duration of HPS, ranging from 7.2 to 97.6 months [6,18,22,24,29,32,33,34,35,43,46,47,49,53,54,58,59,61,63,64,68,69,71,72]. The study period investigated by the included studies is presented in Supplementary Figure S2. The mean duration of the included studies was 17 years (range 1–45 years).

### 3.3. Mortality

**Figure 2:**
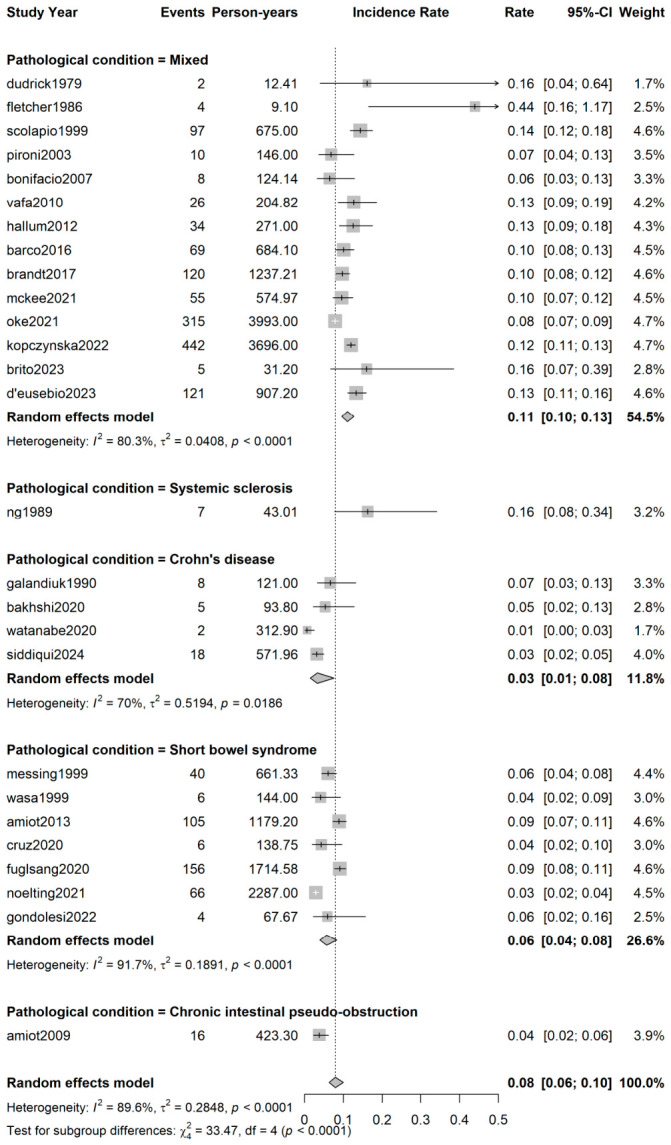
Incidence rates of death per year reported by included studies, shown by pathological condition and in ascending order by year of publication. The horizontal lines represent confidence intervals, with arrows indicating extensions of the intervals. Boxes represent estimated mortality rates, with the sizes of the boxes indicating the inverse variance weight of the respective studies. Diamonds represent the pooled mortality rate. The width of the diamonds represents the width of the 95% confidence interval of the pooled mortality rate [6,18,19,20,22,25,29,31,32,33,38,40,43,47,51,54,55,58,64,65,66,67,68,69,71,72,74].

Overall, 27 studies reported a number of deaths over a mean follow-up period [6,18,19,20,22,25,29,31,32,33,38,40,43,47,51,54,55,58,64,65,66,67,68,69,71,72,74]. In all of the studies, patient baseline enrolment was at the time of starting HPS. The overall mortality of patients with chronic IF was 8.0% per year of follow-up (95% CI 6.0 to 10.0%) (Figure 2), or 33.5% (1747/5212) over a mean of 49.02 months of follow-up, with high heterogeneity between studies (I^2^ = 89.6%, *p* < 0.001). The mortality in patients from mixed populations was 11.0% per year of follow-up (95% CI 10.0% to 13.0%, I^2^ = 80.3%, *p* < 0.001), in patients with chronic IF due to Crohn’s disease it was 3.0% per year (95% CI 1.0% to 8.0%, I^2^ = 70.0%, *p* = 0.02), and in patients with chronic IF due to SBS it was 6.0% per year (95% CI 4.0% to 8.0%, I^2^ = 91.7%, *p* < 0.001) (Figure 2). Subgroup analyses of overall mortality by centre size, type of follow-up, and reported risk of bias in studies are presented in Supplementary Figure S3.

**Figure 3:**
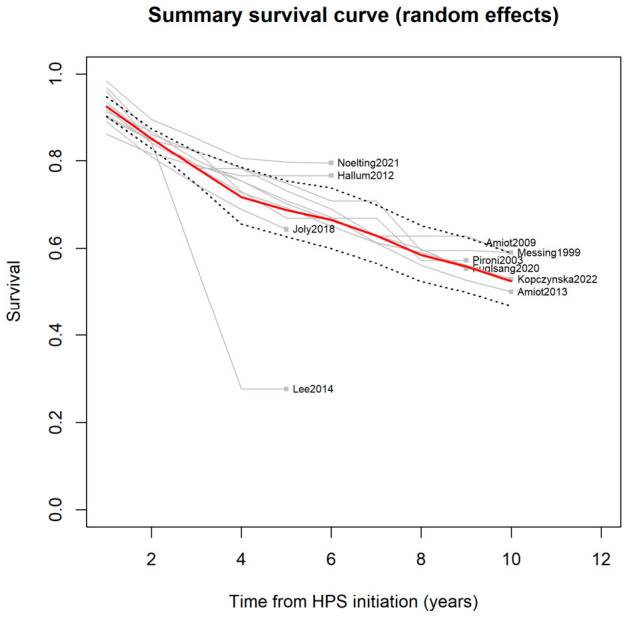
Curves of the overall survival for patients with benign intestinal failure on home parenteral support in the 10 studies included in the meta-analysis. Notes: The grey lines represent the survival in each study, and the grey square the end of the follow-up. Each of the survival curves is labelled with the first author’s name and year of publication. The thick red line represents the summarised survival curves, with the 95% confidence bands (dashed lines) obtained with random effects [18,20,22,38,43,54,55,56,62,68].

Twelve studies presented survival curves with follow-up from the initiation of HPS to end of the follow-up, ranging from a maximum of five to forty years [18,20,22,38,43,53,54,55,56,62,68,69]. Two studies were excluded from the analysis due to unreadability of the survival curves [53,69]. The summary of the survival probabilities over time is presented in Supplementary Table S2 and Figure 3. The decline in survival was more pronounced during the initial five years of follow-up (~6–7% mortality per year) compared to the following years (~2–4% mortality per year). Overall, three studies followed patients until HPS cessation [43,62,68]; in the rest of the studies, patients were followed up until death or the end of the study period [18,20,22,38,54,55,56].

### 3.4. Factors Associated with Mortality

Due to a small number of studies investigating potential risk factors using the same definitions and methods, a meta-regression of factors associated with mortality was not possible. The summary of factors with positive and negative association reported by the included studies is presented in Supplementary Figure S4. A frequently reported factor associated with mortality was older age, which was identified as a significant risk factor in thirteen studies [18,19,20,22,24,37,38,40,43,55,62,72,75]. Seven studies applied age cut-offs for the analysis (e.g., age ≥ 60 years) [9,20,22,24,37,38,62], and six studies used age as a continuous variable [18,19,40,43,72,75]. Another frequently considered risk factor was gastrointestinal anatomy. Shorter small bowel lengths were reported to be associated with increased mortality in five studies [37,38,40,65,72]. The presence of colon-in-continuity (one study [19]) and restoration of gastrointestinal tract continuity (two studies [65,72]) were reported to be negatively associated with mortality. The associations between the pathological conditions leading to chronic IF and mortality were also evaluated by several studies. The most frequently reported factors associated with increased mortality among the underlying diseases leading to chronic IF were mesenteric ischaemia (eight studies [9,18,19,22,38,54,55,72]), systemic sclerosis (two studies [20,76]), and radiation enteritis (two studies [18,19]). Crohn’s disease leading to chronic IF was associated with reduced mortality in three studies [37,62,76]. Regarding the mechanisms leading to chronic IF, SBS-group 1 (two studies [38,55]), chronic obstruction (two studies [9,19]), and fistulae (two studies [9,19]) were reported to be associated with increased mortality. Long-term HPS dependence was also associated with increased mortality in four studies [18,22,38,55]. Other identified factors presented in Supplementary Figure S4 were reported by single studies.

### 3.5. Causes of Death

Cause of death was reported in 34 studies [9,18,20,24,26,29,31,32,34,37,38,39,40,43,44,45,46,47,48,49,51,52,55,61,62,63,66,67,68,69,73,79,80]. The main causes of death were non-IF/non-HPS-related (45.6%), followed by underlying disease (35.2%) and IF-related death (12.2%); 7.1% of deaths had an unknown cause. Overall, 5.1% of deaths were due to catheter-related bloodstream infection, 0.5% due to central venous catheter thrombosis, 3.9% due to IF-associated liver disease, and 2.5% due to unspecified IF-related causes.

### 3.6. Nutritional Autonomy

**Figure 4:**
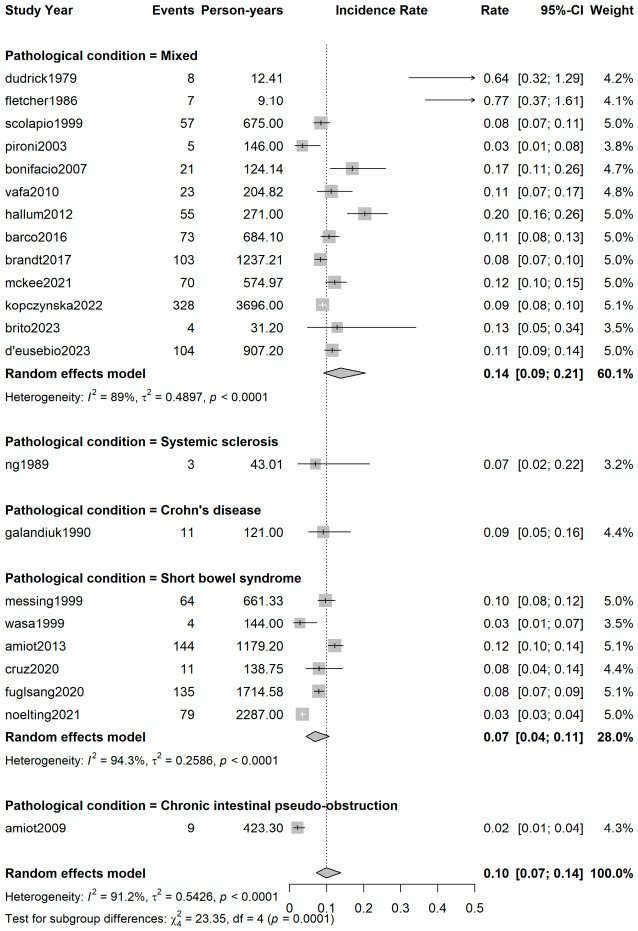
Incidence rates of regaining nutritional autonomy reported by included studies, shown by pathological condition in ascending order of publication. The horizontal lines represent confidence intervals, with arrows indicating extensions of the intervals. Boxes represent estimated autonomy rates, with the sizes of the boxes indicating the inverse variance weight of the respective studies. Diamonds represent the pooled autonomy rate. The width of the diamonds represents the width of the 95% confidence interval of the pooled autonomy rate [6,18,20,22,25,29,31,32,33,38,40,43,47,51,54,55,58,65,67,68,71,72].

Overall, 22 studies reported the number of patients who regained nutritional autonomy over a mean follow-up period [6,18,20,22,25,29,31,32,33,38,40,43,47,51,54,55,58,65,67,68,71,72]. Nutritional autonomy was achieved in 35.3% (1318/3736) of patients, at a rate of 10.0% per year of follow-up (95% CI 7.0% to 14.0%), with high heterogeneity between studies (I^2^ = 91.2%, *p* < 0.001) (Figure 4). In mixed populations of patients with chronic IF, nutritional autonomy was achieved at rate of 14.0% per year of follow-up (95% CI 9.0% to 21.0%, I^2^ = 89.0%, *p* < 0.001), and in patients with chronic IF due to SBS at 7.0% per year (95% CI 4.0% to 11.0%, I^2^ = 94.3%, *p* < 0.001) (Figure 4). Overall, only three studies [22,72,77] used death as a competing risk in the analysis of the regaining nutritional autonomy rate. Subgroup analyses of regaining nutritional autonomy by centre size, type of analysis, and reported risk of bias in studies are presented in Supplementary Figure S5.

### 3.7. Factors Associated with Regaining Nutritional Autonomy

The challenges in analysing the factors associated with nutritional autonomy were similar to those described in the mortality section; thus, only the summary of factors with positive and negative associations reported by the included studies is presented (Supplementary Figure S6). The most frequently reported factors associated with regaining nutritional autonomy were based on gastrointestinal anatomy. Increased length of the small bowel was associated with increased likelihood of regaining nutritional autonomy in five studies [38,52,72,77,78]. Additionally, increased length of the colon (two studies [72,78]), colon-in-continuity (two studies [77,78]), ileocaecal valve presence (one study [65]), and reconstructive surgery after HPS initiation (one study [72]) were reported to be associated with increased likelihood of nutritional autonomy. Regarding the mechanisms leading to chronic IF, SBS-group 1 and SBS-group 2 were negatively associated with achieving nutritional autonomy, in contrast to SBS-group 3 in three studies [22,38,72]. Moreover, chronic intestinal pseudo-obstruction (CIPO) was associated with decreased likelihood of nutritional autonomy in two studies [20,62]. Older age at HPS initiation was also reported to be associated with decreased likelihood of achieving nutritional autonomy in three studies [9,72,77]. Other identified factors presented in Supplementary Figure S6 were reported by single studies.

### 3.8. Risk of Bias in Studies

The Risk of Bias in Non-Randomised Studies of Exposures tool was used to assess the risk of bias in the included studies. The detailed appraisal is presented in Supplementary Figure S7. In terms of overall quality, 17 studies had a low risk of bias [9,18,27,33,34,35,40,47,52,57,58,59,63,66,67,71,72], 32 studies had some concerns of bias [6,19,22,24,25,29,30,31,32,37,38,39,42,43,44,45,46,48,49,53,54,55,56,62,64,65,68,69,70,74], and nine studies had a high risk of bias [26,36,41,50,51,60,61,73]. Some concerns were raised regarding confounding, as few studies included detailed assessment of its impact on the measured outcomes. Some concerns were raised due to temporal bias in relation to exposure to HPS, as it is likely that not all patients were treated with the same HPS preparations over time. Concerns were also raised regarding censoring, as patients could have been censored upon death, the end of the study date, ceasing HPS, or receiving alternative treatment such as a small bowel transplant. Some concerns were raised with the reporting of missing data, with few studies reporting external checks on validity. Additionally, no study protocols were available to assess selection bias in reporting.

## 4. Discussion

This study presents the first systematic review of the current literature on survival and the ability to regain nutritional autonomy in HPS-dependent adults with chronic IF, with data assembled from 57 studies. The included studies were from fifteen different countries as well as international collaborations across Europe and worldwide. Patients were followed up from as early as 1969 [31], with most studies following the patient cohorts up over a few decades. Uniquely in this patient group, we present the data in a meta-analysis, providing rates for the two most important clinical outcomes in chronic IF. These findings, together with a thorough overview of risk factors identified by the included studies, increase our understanding of this complex condition and provide useful baseline data for clinical decision-making and patient counselling.

Our meta-analysis shows that the overall mortality of patients with chronic IF was 8.0% per year of follow-up (95% CI 6.0 to 10.0%), or 33.5% over a mean of 49.02 months of follow-up. Notably, studies of patients with chronic IF due to Crohn’s disease [33,64,66,74] had substantially lower mortality rates in comparison to studies with mixed populations [6,18,19,25,32,40,43,47,51,54,58,67,71,72]; these are important data for patients living with inflammatory bowel disease, as well as for their treating clinicians, who can often fear HPS-dependency as representing an end-stage process of their disease [66]. The summary of survival curves estimated the pooled five- and ten-year survival rates from HPS initiation as 68.71% and 52.46%, respectively, with a more pronounced decline in survival during the initial five years of follow-up in comparison to the following years. Studies from Europe and North America had similar mortality rates over time [9,18,20,22,38,54,55,62,68], in contrast to the study conducted in Brazil [56], with a substantially higher mortality rate, which was suggested by the study authors to be due to the low socio-economic income and low literacy rates of patients, which made HPS management challenging, reiterating the need for tailored and focussed education for patients living with this condition [81]. Moreover, while in our pooled results only 12.2% of patient deaths were deemed to be IF-related, with non-IF/non-HPS-related causes reported as the primary reason in 45.6% of patients, it is noteworthy that over 5% of all deaths were related to catheter-related bloodstream infections, highlighting the need for sustained educational approaches in aseptic technique and promotion of the use of novel anti-microbial locks to mitigate this complication [81].

Out of the studies included in the survival curve summary analysis, three followed patients until HPS weaning [43,62,68], with the rest following patients until death or the end of the study period [18,20,22,38,54,55,56]. The type of follow-up is important, as the former method violates the independent censoring assumption, since such a model is a competing risk model. This can lead to an underestimation of the true survival rate due the fact that the least frail patients are more likely to wean off HPS and, hence, are censored by the Kaplan–Meier method in the survival function. In order to adjust for that, Fuglsang et al., in their landmark paper, suggested performing analysis with the Aalen–Johansen estimator—a method appropriate for estimating cumulative incidence in case of competing risks in cohorts followed up until HPS cessation—and ensuring that the results are interpreted as “cumulative mortality before attempted weaning off” [22] when making comparisons between studies.

Moreover, our meta-analysis showed that nutritional autonomy was achieved in 35.3% of patients, at a rate of 10.0% per year of follow-up (95% CI 7.0% to 14.0%). Studies on patients with chronic IF due to SBS [22,31,38,55,65,68] had a substantially lower rate of regaining nutritional autonomy in comparison to studies with mixed populations [6,18,25,32,40,43,47,51,54,58,67,71,72], likely because of surgical or medical interventions available for other chronic IF disease mechanisms—such as surgical intervention for fistula repair or alleviation of obstruction—that facilitate HPS weaning. Overall, only three studies [22,72,77] used death as a competing risk in the analysis of the nutritional autonomy rate and constructed cumulative incidences curves using the Aalen–Johansen estimator, with the rest of the studies providing results based on Kaplan–Meier estimates. The problem with the latter method is that the obtained estimates should in fact be interpreted as the “probability of HPS dependence in immortal patients” because deaths are censored [22]. It clearly only makes sense to investigate HPS cessation before death; thus, the competing risk analysis provides less biased results and should be implemented in the analysis whenever possible.

In regards to both mortality and nutritional autonomy, the meta-regression of associations was not possible due to the limited number of studies investigating potential risk factors using the same definitions and methodology. However, in summary, age, gastrointestinal anatomy, and pathological conditions leading to IF were identified by multiple studies as potential factors associated with overall mortality and nutritional autonomy. Long-term HPS dependence has also been reported to be associated with increased mortality; however, it is important to assess such analysis for an immortal time bias, where HPS-independent patients cannot die before they become independent, and so it is vital to use this variable as a time-varying covariate in the analysis. Overall, these results emphasise the inconsistency in the reported risk factors associated with clinical outcomes among chronic IF outcome studies and highlight the need for standard outcome reporting measures in research that can then be used to develop validated clinical prediction models for patients with different disease aetiologies leading to chronic IF.

The findings of this study have significant implications for routine clinical practice, as well as for future chronic IF service developments. Extracting and synthesising global data on long-term outcomes of HPS-dependent patients with chronic IF has emphasised the burden of this disorder and, thus, has implications for health services worldwide. We have shown that long-term outcomes in this patient cohort can be good. This might encourage policymakers to invest resources to establish appropriate services in countries in currently unable to provide HPS [82].

Furthermore, monitoring of outcomes is essential to achieve clinical and patient-centred improvement, which is why it has been recommended as a quality-of-care standard in the management of patients with chronic IF by the European Society of Clinical Nutrition and Metabolism (ESPEN) in their recent position paper [8]. Indeed, healthcare professionals providing care to patients with chronic IF could be encouraged to set tangible outcome targets that are specific to patient needs based on a review of their current practice and collection of baseline data, whether within an individual centre, across a wider region, or via a national or international network of chronic IF centres, using the same methodology for patient classification and reporting. This meta-analysis, with sub-analysis of pathological conditions leading to IF, could therefore enable centres to benchmark their patient outcomes. On a patient level, better prediction of patient survival and/or nutritional autonomy rates could lead to more informed decision-making on continuation with HPS vs. implementing alternative treatments such as glucagon-like peptide 2 analogues, autologous gastrointestinal reconstruction, or small bowel transplant (for example, early proactive selection of patients with a shorter remnant small bowel or SBS-group 1 for such interventions). Additionally, better prediction of the likelihood of nutritional autonomy could help with stratification of patients in whom more caution is required in the assessment and mitigation of further complications associated with HPS, such as catheter-related bloodstream infection, central venous catheter thrombosis, or IF-associated liver disease. Equally, better patient prognostication could also be helpful during patient consultations to manage their expectations and address concerns.

This study has several strengths. We used a broad search strategy in order to maximise the likelihood of identifying all pertinent literature. The assessment of study eligibility was carried out by two investigators independently, with discrepancies resolved by consensus with a third investigator. Moreover, we included studies carried out in cohort or cross-sectional studies and excluded studies reporting outcomes in clinical trials; thus, the presented data should be generalisable to patients from both specialist as well as less established chronic IF centres. However, this meta-analysis was limited by the fact that, as a cohort meta-analysis, it was not possible to follow individuals through their disease course. If individual patient-level data had been available, this would have allowed a more detailed analysis of patients’ disease course by specific characteristics such as age, sex, pathological condition leading to chronic IF, or year of HPS initiation. Indeed, trends in improvements in clinical outcomes over time could not be assessed, as most studies provided outcomes for patients over a few decades, with the longest study following patients up for 45 years [22]. Moreover, there was significant heterogeneity between studies in our analyses. Due to the lack of detailed patient data preventing extensive subgroup analyses, the reasons for the heterogeneity are therefore speculative and may include differences between study populations including patient demographics and clinical characteristics, as well as access to novel treatments and quality of standard of care received by the patients. Indeed, subgroup analyses based on patient pathophysiological condition showed differences in the pooled overall mortality and nutritional autonomy. Furthermore, a risk of cohort meta-analyses is the potential for double-counting of individual patients by their inclusion in more than one study; however, we attempted to limit this by excluding data from overlapping cohorts at each centre. The risk-of-bias analysis raised concerns over the assessment of confounders and reporting of missing data, which are frequent issues with retrospective studies. In order to reduce this bias, future studies should strive to carry out prospective data collection with a protocol developed *a priori*, ideally in a multicentre setting or an international patient registry.

## 5. Conclusions

In conclusion, this study presents the first systematic review and meta-analysis on survival and the ability to regain nutritional autonomy in adults with chronic IF, which has generated rates for clinical outcomes. These findings increase our understanding of this condition, provide useful baseline data for clinical decision-making and patient counselling, and provide a list of candidate predictors for clinical prediction model development. From a methodological perspective, this study highlights the importance of using a survival and nutritional autonomy estimation methodology that results in reduced bias, such as by following patients until death or the end of the study period for survival analysis, and using death as a competing risk when analysing nutritional autonomy in HPS-dependent individuals.

## Figures and Tables

**Table 1:** Study and participant characteristics of included studies on adult patients with benign chronic intestinal failure receiving home parenteral support.

Name of Author, Year of Publication	Centre	Country	Study Years from -> to	Inclusion Criteria	Exclusion Criteria	Study Design	Single/ Multicentre	Sample Size	Mean Age (Years)	Proportion of Male Patients	Follow-Up	Median HPS Duration	Range of HPS Duration
Dudrick 1979 [25]	Hermann Hospital	USA		Patients with CIF	NA	Retrospective cohort study	Single	25			4530 person-days		
Fletcher 1986 [32]	Westmead Hospital	Australia	1979 -> 1986	Patients with CIF	NA	Retrospective cohort study	Single	13	42.4		9.1 person-years	255 days	14–608 days
Messing 1989 [28]	Multicentre	Europe	1974 -> 1985	Patients with CIF	NA	Retrospective cohort study	Multi	194	44	52.1%			
Ng 1989 [29]	UCLA Hospital	USA	1979 -> 1987	Patients with CIF due to scleroderma/CREST	NA	Retrospective cohort study	Single	15		6.7%	15,700 person-days	2.9 years	2 months–7.5 years
Galandiuk 1990 [33]	Cleveland Clinic	USA	1976 -> 1987	Patients with CIF due to Crohn’s disease	NA	Retrospective cohort study	Single	41	39	39.0%	121 person-years	1083 days	33–3258 days
Howard 1991 [26]	OASIS Registry	USA	1984 -> 1987	Patients with CIF	NA	Retrospective cohort study	Multi	1594					
O’hanrahan 1992 [34]	Multicentre	UK	1977 -> 1991	Patients with CIF	NA	Retrospective cohort study	Multi	400		52.5%		11.2 months	1–77 months
Johnston 1993 [27]	Ninewells Hospital and Medical School	UK	1980 -> 1992	Patients with CIF	NA	Prospective cohort study	Single	30		56.7%	17,327 person-days		
Takagi 1995 [35]	Multicentre	Japan	1990	Patients with CIF	NA	Retrospective cross-sectional study	Multi	231	46	60.6%		683 days	
Shields 1996 [30]	University Hospital Queen’s Medical Centre	UK	1983 -> 1993	Patients with CIF	NA	Retrospective cohort study	Single	162			4997 person-days		
Van Gossum 1996 [36]	Multicentre	Europe	1993 -> 1994	Patients with CIF	NA	Retrospective cohort study	Multi	496		11.5%			
Jeppesen 1998 [37]	Multicentre	Denmark	1991 -> 1995	Patients with CIF	NA	Retrospective cohort study	Multi	129		36.4%	313.5 person-years		
Messing 1999 [38]	Hopital Lariboisiere-St. Lazare/Hopital La Miletrie	France	1980 -> 1992	Patients with CIF due to SBS	Severe visceral failure at the time of short bowel occurrence; or patients with evolving primary malignancy either present at the time of short bowel occurrence or recurring during follow-up; or patients who had received other treatments than HPN for intestinal failure, e.g., growth hormone or reconstructive surgery of the remnant bowel, such as surgically reversed small bowel loop or small bowel transplantation	Retrospective cohort study	Multi	124	52	50.0%			
Reimund 1999 [39]	Hopitaux Universitaires de Strasbourg	France	1994 -> 1997	Patients with CIF	NA	Retrospective cohort study	Single	27	55.7	70.4%			
Scolapio 1999 [40]	Mayo Clinic	USA	1975 -> 1995	Patients with CIF	NA	Retrospective cohort study	Single	225	51	46.2%			
Van Gossum 1999 [41]	Multicentre	Europe	1997 -> 1998	Patients with CIF	NA	Retrospective cohort study	Multi	494		41.1%			
Wasa 1999 [31]	Osaka University Hospital	Japan	1969 -> 1999	Patients with CIF due to SBS	NA	Retrospective cohort study	Single	18		44.4%			
Cavicchi 2000 [24]	Hopital Lariboisiere-St. Lazare/Hopital La Miletrie	France	1985 -> 1996	Patients with CIF	Malignancy, AIDS	Prospective cohort study	Multi	90	45	55.6%		45 months	6–198 months
Thompson 2000 [42]	Omaha Veterans Administration Medical Center and University of Nebraska Medical Center	USA	1980 -> 1999	Patients with CIF due to SBS	NA	Retrospective cohort study	Single	106					
Pironi 2003 [43]	Chronic Intestinal Failure Centre of Bologna University	Italy	1986 -> 2001	Patients with CIF	Malignancy, AIDS	Retrospective cohort study	Single	40	42.6	45.0%	146 person-years	3.74 years	0.08–11.02 years
Freshwater 2005 [44]	Russells Hall Hospital	UK	1989 -> 2002	Patients with CIF	NA	Retrospective cohort study	Single	23	50	43.5%	16,173 person-days		
Moreno-Villares 2006 [45]	Multicentre	Spain	1992 -> 2003	Patients with CIF	NA	Retrospective cohort study	Multi	86	51	38.4%			
Ugur 2006 [46]	Multicentre	Denmark	1996 -> 2001	Patients with CIF	NA	Retrospective cohort study	Multi	202	54	43.1%		5.1 years	30 days–32 years
Bonifacio 2007 [47]	Naples/Rotondo	Italy	1995 -> 2005	Patients with CIF	HPN < 6 months, oncology patients	Retrospective cohort study	Multi	41	52	48.8%	35,449 person-days	630 days	77–2705 days
Marra 2007 [48]	The Virginia Commonwealth University Medical Center	USA	1981 -> 2005	Patients with CIF	HPN < 6 months	Retrospective cohort study	Single	47	44.8	29.8%			
Green 2008 [49]	John Radcliffe Hospital	UK	1990 -> 2004	Patients with CIF	NA	Retrospective cohort study	Single	88	40	45.5%	121 person-years	217.8 days	
Amiot 2009 [20]	Hopital Lariboisiere-St. Lazare/Beaujon Hospital	France	1980 -> 2006	Patients with CIF due to CIPO	Exclusion criteria were (a) chronic organic intestinal obstruction, such as peritoneal carcinosis, radiation enteritis, and Crohn’s disease; and (b) CIPO patients without HPN requirement	Retrospective cohort study	Multi	51	20	35.3%			
Putchakayala 2009 [50]	Northwestern Memorial Hospital	USA	1999 -> 2006	Patients with CIF	NA	Retrospective cohort study	Single	133	50.7	36.8%			
Vafa 2010 [51]	Hopital Erasme, Universit? Libre de Bruxelles	Belgium	1987 -> 2007	Patients with CIF	NA	Retrospective cohort study	Single	65	52	46.2%	74,760 person-days		1–240 months
Elriz 2011 [52]	Multicentre	France	1986 -> 2004	Patients with CIF due to Crohn’s disease	HPN < 12 months	Retrospective cohort study	Multi	38		52.6%			
Guglielmi 2012 [53]	Gastroenterology Unit of the University of Bari/Gastroenterology Unit of San Pellegrino Hospital	Italy	1984 -> 2009	Patients with CIF	NA	Retrospective cohort study	Multi	131				830 days	
Hallum 2012 [54]	Scottish HPN Managed Clinical Network	UK	2000 -> 2007	Patients with CIF	NA	Prospective cohort study	Multi	136	47	33.8%	271 person-years	2 years	1 month–8 years
Amiot 2013 [55]	Beaujon Hospital	France	1980 -> 2006	Patients with CIF due to SBS	HPN < 3 months, malignancy	Retrospective cohort study	Single	268	52.5	48.1%			
Lee 2014 [56]	Department of Gastroenterology University of Sao Paulo	Brazil	1991 -> 2013	Patients with CIF	NA	Retrospective cohort study	Single	128		60.2%			
Watanabe 2014 [57]	Multicentre	Japan	1970 -> 2009	Patients with CIF due to Crohn’s disease	NA	Retrospective cohort study	Multi	101	38.2				
Barco 2016 [58]	Academic Medical Center Amsterdam	Netherlands	1986 -> 2014	Patients with CIF	HPN < 3 months, peripheral catheters, incomplete medical data	Retrospective cohort study	Single	236	54	39.8%	684.1 person-years	17 months	
Brandt 2017 [6]	Rigshospitalet	Denmark	1970 -> 2010	Patients with CIF	NA	Retrospective cohort study	Single	508	53.9	41.7%	1750.7 person-years	519 days	2–12,414 days
Lorentsen 2017 [59]	Zealand University Hospital	Denmark	2005 -> 2014	Patients with CIF due to SBS	NA	Retrospective cohort study	Single	78	64			860 days	
Van Gossum 2017 [60]	Multicentre	Europe	2008 -> 2014	Patients with CIF due to post-bariatric complications	NA	Retrospective cohort study	Multi	77	50	15.6%			
Wu 2017 [61]	Department of General Surgery Zhongshan Hospital of Fudan University	China	1985 -> 2015	Patients with CIF due to SBS	HPN < 2 years, malignancy	Retrospective cohort study	Single	47	45.1	44.7%		8.13 years	
Joly 2018 [62]	Multicentre	Europe/USA	2000 -> 2004	Patients with CIF	Malignancy, AIDS	Retrospective cohort study	Multi	472	51	42.2%			
Koelfat 2019 [63]	Department of Gastroenterology of Radboud University Medical Center	Netherlands	2007 -> 2017	Patients with CIF	HPN < 3 months, no clinical data and blood samples; patients with obstructive cholestasis caused by biliary malignancies or autoimmune diseases [e.g., primary sclerosing cholangitis]	Prospective cohort study	Single	135	50.3	25.9%		46 months	14–83 months
Bakhshi 2020 [64]	Olmsted Medical Center	USA	1992 -> 2018	Patients with CIF due to Crohn’s disease	NA	Retrospective cohort study	Single	14		14.3%		2.4 years	40 days–16.4 years
Cruz 2020 [65]	University of Pittsburgh Medical Center	USA	2013 -> 2018	Patients with CIF due to SBS	SB > 50 cm	Retrospective cohort study	Single	45	46.7	42.2%			
Fuglsang 2020 [22]	Rigshospitalet	Denmark	1970 -> 2015	Patients with CIF due to SBS	IF due to another mechanism, SB > 200	Retrospective cohort study	Single	331	54.9		2429 person-years	1.65 years	
Pironi 2020 [9]	Multicentre	Worldwide	2015 -> 2016	Patients with CIF	Malignancy	Retrospective cohort study	Multi	2194	51.1	37.0%			
Watanabe 2020 [66]	Osaka University Hospital	Japan	2000 -> 2019	Patients with CIF due to Crohn’s disease	NA	Retrospective cohort study	Single	21	39	61.9%			
McKee 2021 [67]	Greater Glasgow and Clyde Health Board	UK	1993 -> 2018	Patients with CIF	NA	Retrospective cohort study	Multi	205	56	38.0%	209,864 person-days		
Noelting 2021 [68]	Canadian HPN Registry	Canada	1972 -> 2017	Patients with CIF due to SBS	NA	Prospective cohort study	Multi	321		34.9%	2287 person-years	4.2 years	
Oke 2021 [19]	St Mark’s Hospital	UK	1979 -> 2016	Patients with CIF	Patients with short-term IF	Retrospective cohort study	Single	978	52.4	42.0%	3993 person-years		
Gondolesi 2022 [69]	Multicentre	Argentina	2017 -> 2020	Patients with CIF due to SBS	Incomplete written consent	Prospective cohort study	Multi	56	50	48.2%		33.5 months	
Kopczynska 2022 [18]	Salford Royal Hospital	UK	1978 -> 2018	Patients with CIF	NA	Retrospective cohort study	Single	1046	53	40.2%	7344.1 person-years	2.8 years	0.02–37.9 years
Thompson 2022 [70]	University of Nebraska Medical Center	USA	1990 -> 2019	Patients with CIF due to SBS	Follow-up < 12 months, patients with cholecystectomy after developing SBS	Retrospective cohort study	Single	485					
Brito 2023 [71]	Hospital Garcia de Orta	Portugal	2011 -> 2021	Patients with CIF	NA	Retrospective cohort study	Single	13	63.46	46.2%		23 months	
D’Eusebio 2023 [72]	Citta della Salute e della Scienza Hospital of Torino	Italy	1985 -> 2016	Patients with CIF	HPN < 3 months, active neoplastic disease and/or being under anti-neoplastic treatment within the previous 5 y, inability to give informed consent, and critically ill patients with a <6-month life expectancy	Retrospective cohort study	Single	324	57.2	45.4%	1524 person-years	2.8 years	
Santos 2024 [73]	Centro Hospitalar Universitario de Santo Antonio	Portugal	1994 -> 2023	Patients with CIF due to SBS	Type 2 IF, death prior to discharge on HPN	Retrospective cohort study	Single	13	44	38.5%			
Siddiqui 2024 [74]	Cleveland Clinic HPN Registry	USA	2013 -> 2019	Patients with CIF due to IBD	NA	Retrospective cohort study	Single	407	45.7	46.4%	208,767 person-days		

Notes: Blank cells correspond to data not being provided in studies. CIF, chronic intestinal failure; CIPO, chronic intestinal pseudo-obstruction; IBD, inflammatory bowel disease; HPS, home parenteral support; SBS, short bowel syndrome.

## Data Availability

The original contributions presented in this study are included in the article/Supplementary Materials. Further inquiries can be directed to the corresponding author.

## Supplementary Materials

The following supporting information can be downloaded at https://www.mdpi.com/article/10.3390/nu18132123/s1, Supplementary Table S1. The PRISMA 2020 checklist; Supplementary Table S2. Pooled estimates of the survival probabilities from 10 studies included in the meta-analysis for patients with benign intestinal failure on home parenteral support; Supplementary Figure S1. Search strategies for all databases; Supplementary Figure S2. The time period for the included studies in years from 1969 to 2023; Supplementary Figure S3. Sub-group analysis of the overall mortality; Supplementary Figure S4. Previously reported associations of all-cause mortality from studies included in the systematic review; Supplementary Figure S5. Sub-group analysis of the regaining nutritional autonomy; Supplementary Figure S6. Previously reported associations of regaining nutritional autonomy from studies included in the systematic review. Supplementary Figure S7. Risk-of-bias chart for studies included in the systematic review.

## Abbreviations

The following abbreviations are used in this manuscript:

IF	Intestinal failure
HPS	Home parenteral support
CIPO	Chronic intestinal pseudo-obstruction
SBS	Short bowel syndrome

## References

[1] Pironi L., Arends J., Baxter J., Bozzetti F., Peláez R.B., Cuerda C., Forbes A., Gabe S., Gillanders L., Holst M. (2015). ESPEN endorsed recommendations. Definition and classification of intestinal failure in adults. Clin. Nutr..

[2] Bines J.E. (2009). Intestinal failure: A new era in clinical management. J. Gastroenterol. Hepatol..

[3] Klek S., Forbes A., Gabe S., Holst M., Wanten G., Irtun Ø., Damink S.O., Panisic-Sekeljic M., Pelaez R.B., Pironi L. (2016). Management of acute intestinal failure: A position paper from the European Society for Clinical Nutrition and Metabolism (ESPEN) Special Interest Group. Clin. Nutr..

[4] Pironi L., Cuerda C., Jeppesen P.B., Joly F., Jonkers C., Krznarić Ž., Lal S., Lamprecht G., Lichota M., Mundi M.S. (2023). ESPEN guideline on chronic intestinal failure in adults—Update 2023. Clin. Nutr..

[5] Rhoda K.M., Parekh N.R., Lennon E., Shay-Downer C., Quintini C., Steiger E., Kirby D.F. (2010). The multidisciplinary approach to the care of patients with intestinal failure at a tertiary care facility. Nutr. Clin. Pract..

[6] Brandt C.F., Hvistendahl M., Naimi R.M., Tribler S., Staun M., Brobech P., Jeppesen P.B. (2017). Home Parenteral Nutrition in Adult Patients with Chronic Intestinal Failure: The Evolution Over 4 Decades in a Tertiary Referral Center. J. Parenter. Enter. Nutr..

[7] Wang S.Z., O’Daniel E.L. (2025). Updates in Intestinal Failure Management. J. Clin. Med..

[8] Lal S., Soop M., Cuerda C., Jeppesen P., Joly F., Lamprecht G., Mundi M., Szczepanek K., Van Gossum C., Wanten G. (2024). Quality-of-care standards in adult type 3 intestinal failure caused by benign disease: A European society of clinical nutrition and metabolism (ESPEN) position paper. Clin. Nutr. ESPEN.

[9] Pironi L., Steiger E., Joly F., Wanten G.J.A., Chambrier C., Aimasso U., Sasdelli A.S., Szczepanek K., Jukes A., Theilla M. (2020). Intravenous supplementation type and volume are associated with 1-year outcome and major complications in patients with chronic intestinal failure. Gut.

[10] Fuglsang K.A., Brandt C.F., Scheike T., Jeppesen P.B. (2020). Hospitalizations in Patients With Nonmalignant Short-Bowel Syndrome Receiving Home Parenteral Support. Nutr. Clin. Pract..

[11] Arhip L., Serrano-Moreno C., Romero I., Camblor M., Cuerda C. (2021). The economic costs of home parenteral nutrition: Systematic review of partial and full economic evaluations. Clin. Nutr..

[12] Tribler S., Brandt C.F., Hvistendahl M., Staun M., Brøbech P., Moser C.E., Jeppesen P.B. (2018). Catheter-Related Bloodstream Infections in Adults Receiving Home Parenteral Nutrition: Substantial Differences in Incidence Comparing a Strict Microbiological to a Clinically Based Diagnosis. J. Parenter. Enter. Nutr..

[13] Shike M., Harrison J.E., Sturtridge W.C., Tam C.S., Bobechko P.E., Jones G., Murray T.M., Jeejeebhoy K.N. (1980). Metabolic bone disease in patients receiving long-term total parenteral nutrition. Ann. Intern. Med..

[14] Woodward J.M., Massey D., Sharkey L. (2020). The Long and Short of IT: Intestinal failure-associated liver disease (IFALD) in adults—Recommendations for early diagnosis and intestinal transplantation. Frontline Gastroenterol..

[15] Winkler M.F., Hagan E., Wetle T., Smith C., Maillet J.O., Touger-Decker R. (2010). An exploration of quality of life and the experience of living with home parenteral nutrition. J. Parenter. Enter. Nutr..

[16] Blüthner E., Bednarsch J., Stockmann M., Karber M., Pevny S., Maasberg S., Gerlach U.A., Pascher A., Wiedenmann B., Pratschke J. (2020). Determinants of Quality of Life in Patients With Intestinal Failure Receiving Long-Term Parenteral Nutrition Using the SF-36 Questionnaire: A German Single-Center Prospective Observational Study. J. Parenter. Enter. Nutr..

[17] Lloyd D.A., Vega R., Bassett P., Forbes A., Gabe S.M. (2006). Survival and dependence on home parenteral nutrition: Experience over a 25-year period in a UK referral centre. Aliment. Pharmacol. Ther..

[18] Kopczynska M., Hvas C.L., Jepsen P., Teubner A., Abraham A., Burden S.T., Taylor M., Carlson G., Lal S. (2022). Standardised survival and excess Life Years Lost in patients with type 3 intestinal failure. Clin. Nutr..

[19] Oke S.M., Nightingale J.M., Donnelly S.C., Naghibi M., Willsmore J., Lloyd D.A.J., Gabe S.M. (2021). Outcome of adult patients receiving parenteral support at home: 36 years’ experience at a tertiary referral centre. Clin. Nutr..

[20] Amiot A., Joly F., Alves A., Panis Y., Bouhnik Y., Messing B. (2009). Long-Term Outcome of Chronic Intestinal Pseudo-Obstruction Adult Patients Requiring Home Parenteral Nutrition. Am. J. Gastroenterol..

[21] Pironi L., Goulet O., Buchman A., Messing B., Gabe S., Candusso M., Bond G., Gupte G., Pertkiewicz M., Steiger E. (2012). Outcome on home parenteral nutrition for benign intestinal failure: A review of the literature and benchmarking with the European prospective survey of ESPEN. Clin. Nutr..

[22] Fuglsang K., Brandt C., Scheike T., Jeppesen P. (2020). Differences in methodology impact estimates of survival and dependence on home parenteral support of patients with nonmalignant short bowel syndrome. Am. J. Clin. Nutr..

[23] Combescure C., Foucher Y., Jackson D. (2014). Meta-analysis of single-arm survival studies: A distribution-free approach for estimating summary survival curves with random effects. Stat. Med..

[24] Cavicchi M., Beau P., Crenn P., Degott C., Messing B. (2000). Prevalence of liver disease and contributing factors in patients receiving home parenteral nutrition for permanent intestinal failure. Ann. Intern. Med..

[25] Dudrick S.J., Englert D.M., Van Buren C.T., Rowlands B.J., MacFadyen B.V. (1979). New concepts of ambulatory home hyperalimentation. J. Parenter. Enter. Nutr..

[26] Howard L., Heaphey L., Fleming C.R., Lininger L., Steiger E. (1991). Four years of North American registry home parenteral nutrition outcome data and their implications for patient management. J. Parenter. Enter. Nutr..

[27] Johnston D.A., Pennington C.R. (1993). Home parenteral nutrition in Tayside 1980–1992. Scott. Med. J..

[28] Messing B., Landais P., Goldfarb B., Irving M. (1989). Home parenteral nutrition in adults: A multicentre survey in Europe. Clin. Nutr..

[29] Ng S.C., Clements P.J., Berquist W.E., Furst D.E., Paulus H.E. (1989). Home central venous hyperalimentation in fifteen patients with severe scleroderma bowel disease. Arthritis Rheum..

[30] Shields P.L., Field J., Rawlings J., Kendall J., Allison S.P. (1996). Long-term outcome and cost-effectiveness of parenteral nutrition for acute gastrointestinal failure. Clin. Nutr..

[31] Wasa M., Takagi Y., Sando K., Harada T., Okada A. (1999). Long-term outcome of short bowel syndrome in adult and pediatric patients. Nutrition.

[32] Fletcher J., Little J., Mudie J. (1986). Home Parenteral-Nutrition—Westmead-Hospital Experience. Aust. N. Z. J. Surg..

[33] Galandiuk S., O’Neill M., McDonald P., Fazio V.W., Steiger E. (1990). A century of home parenteral nutrition for Crohn’s disease. Am. J. Surg..

[34] O’hanrahan T., Irving M.H. (1992). The role of home parenteral nutrition in the management of intestinal failure—Report of 400 cases. Clin. Nutr..

[35] Takagi Y., Okada A., Sato T., Fukushima T., Shirotani N., Osawa Y., Takeyama H., Taniguchi M., Takehara H., Mizote H. (1995). Report on the first annual survey of home parenteral nutrition in Japan. Surg. Today.

[36] Van Gossum A., Bakker H., De Francesco A., Ladefoged K., Leon-Sanz M., Messing B., Pironi L., Pertkiewicz M., Shaffer J., ESPEN-Home Artificial Nutrition Working Group (1996). Home parenteral nutrition in adults: A multicentre survey in Europe in 1993. Clin. Nutr..

[37] Jeppesen P.B., Staun M., Mortensen P.B. (1998). Adult patients receiving home parenteral nutrition in Denmark from 1991 to 1996: Who will benefit from intestinal transplantation?. Scand. J. Gastroenterol..

[38] Messing B., Crenn P., Beau P., Boutron-Ruault M., Rambaud J., Matuchansky C. (1999). Long-term survival and parenteral nutrition dependence in adult patients with the short bowel syndrome. Gastroenterology.

[39] Reimund J., Duclos B., Cuby C., Malzac D., Zimmermann F., Dietemann J., Beretz L., Baumann R. (1999). Home parenteral nutrition: Clinical and laboratory analysis of initial experience (1994–1997)—Implications for patient management. Ann. Nutr. Metab..

[40] Scolapio J.S., Fleming C.R., Kelly D.G., Wick D.M., Zinsmeister A.R. (1999). Survival of home parenteral nutrition-treated patients: 20 years of experience at the Mayo Clinic. Mayo Clin. Proc..

[41] Van Gossum A., Bakker H., Bozzetti F., Staun M., Leon-Sanz M., Hebuterne X., Pertkiewicz M., Shaffer J., Thul P., ESPEN-Horne Artificial Nutrition Working Group (1999). Home parenteral nutrition in adults: A European multicentre survey in 1997. Clin. Nutr..

[42] Thompson J.S. (2000). Inflammatory disease and outcome of short bowel syndrome. Am. J. Surg..

[43] Pironi L., Paganelli F., Labate A., Merli C., Guidetti C., Spinucci G., Miglioli M. (2003). Safety and efficacy of home parenteral nutrition for chronic intestinal failure: A 16-year experience at a single centre. Dig. Liver Dis..

[44] Freshwater D., Saadeddin A., Deel-Smith P., Digger T., Jones B. (2005). Can home parenteral nutrition be provided by non-specialised centres? 2300 weeks of experience at a district general hospital in the United Kingdom. Clin. Nutr..

[45] Moreno-Villares J., Cuerda C., Planas M., Candela C., León-Sanz M., de Cos A., Pedrón C., Grp N.-S. (2006). Trends in adult home parenteral nutrition in Spain. 1992–2003. Nutr. Hosp..

[46] Ugur A., Marashdeh B.H.S., Gottschalck I., Brobech Mortensen P., Staun M., Bekker Jeppesen P. (2006). Home parenteral nutrition in Denmark in the period from 1996 to 2001. Scand. J. Gastroenterol..

[47] Bonifacio R., Alfonsi L., Santarpia L., Orban A., Celona A., Negro G., Pasanisi F., Contaldo F. (2007). Clinical outcome of long-term home parenteral nutrition in non-oncological patients: A report from two specialised centres. Intern. Emerg. Med..

[48] Marra A., Opilla M., Edmond M., Kirby D. (2007). Epidemiology of bloodstream infections in patients receiving long-term total parenteral nutrition. J. Clin. Gastroenterol..

[49] Green C., Mountford V., Hamilton H., Kettlewell M., Travis S. (2008). A 15-year audit of home parenteral nutrition provision at the John Radcliffe Hospital, Oxford. QJM—Int. J. Med..

[50] Putchakayala K., Polensky S., Fitzhugh J., Cohran V., Buchman A., Fryer J. (2009). An evaluation of the model for end-stage liver disease and serum C-reactive protein as prognostic markers in intestinal failure patients on parenteral nutrition. J. Parenter. Enter. Nutr..

[51] Vafa H., Ballarin A., Arvanitakis M., Vereecken S., Dutat F., Lagasse C., Lievin V., Van Gossum A. (2010). Lessons from a 20 year experience of Home Parenteral Nutrition in adult patients. Acta Gastro-Enterol. Belg..

[52] Elriz K., Palascak-Juif V., Joly F., Seguy D., Beau P., Chambrier C., Boncompain M., Fontaine E., Laharie D., Savoye G. (2011). Crohn’s disease patients with chronic intestinal failure receiving long-term parenteral nutrition: A cross-national adult study. Aliment. Pharmacol. Ther..

[53] Guglielmi F.W., Regano N., Mazzuoli S., Rizzi M., Fregnan S., Leogrande G., Addante I., Guglielmi A. (2012). Catheter-related complications in long-term home parenteral nutrition patients with chronic intestinal failure. J. Vasc. Access.

[54] Hallum N.S., Tan L.B., Baxter J.P., McKee R.F. (2012). Home parenteral nutrition: Outcome and seven year prospective follow up in a nationwide adult population. e-SPEN J..

[55] Amiot A., Messing B., Corcos O., Panis Y., Joly F. (2013). Determinants of home parenteral nutrition dependence and survival of 268 patients with non-malignant short bowel syndrome. Clin. Nutr..

[56] Lee A.D.W., Galvao F.H.F., Dias M.C.G., Cruz M.E., Marin M., Pedrol C.N., David A.I., Pecora R.A.A., Waitzberg D.L., D’Albuquerque L.A.C. (2014). Home parenteral nutrition program and referral of potential candidates for intestinal and multivisceral transplantation in a single Brazilian center. Transplant. Proc..

[57] Watanabe K., Sasaki I., Fukushima K., Futami K., Ikeuchi H., Sugita A., Nezu R., Mizushima T., Kameoka S., Kusunoki M. (2014). Long-term incidence and characteristics of intestinal failure in Crohn’s disease: A multicenter study. J. Gastroenterol..

[58] Barco S., Heuschen C.B.B.C.M., Salman B., Brekelmans M.P.A., Serlie M.J., Middeldorp S., Coppens M. (2016). Home parenteral nutrition-associated thromboembolic and bleeding events: Results of a cohort study of 236 individuals. J. Thromb. Haemost..

[59] Lorentsen R., Munck L.K., Wildt S. (2017). Parenteral therapy and complications in patients with intestinal failure in a regional unit. Scand. J. Gastroenterol..

[60] Van Gossum A., Pironi L., Chambrier C., Dreesen M., Brandt C., Santarpia L., Joly F. (2017). Home parenteral nutrition (HPN) in patients with post-bariatric surgery complications. Clin. Nutr..

[61] Wu G., Jiang Y., Zhu X., Jin D., Han Y., Han J., Wu Z., Wu Z. (2017). Prevalence and risk factors for complications in adult patients with short bowel syndrome receiving long-term home parenteral nutrition. Asia Pac. J. Clin. Nutr..

[62] Joly F., Baxter J., Staun M., Kelly D., Hwa Y., Corcos O., De Francesco A., Agostini F., Klek S., Santarpia L. (2018). Five-year survival and causes of death in patients on home parenteral nutrition for severe chronic and benign intestinal failure. Clin. Nutr..

[63] Koelfat K.V.K., Huijbers A., Schaap F.G., van Kuijk S.M.J., Lenicek M., Soeters M.R., Wanten G.J.A., Olde Damink S.W.M. (2019). Low circulating concentrations of citrulline and FGF19 predict chronic cholestasis and poor survival in adult patients with chronic intestinal failure: Development of a Model for End-Stage Intestinal Failure (MESIF risk score). Am. J. Clin. Nutr..

[64] Bakhshi Z., Yadav S., Salonen B.R., Bonnes S.L., Varayil J.E., Harmsen W.S., Hurt R.T., Tremaine W.J., Loftus E.V.J. (2020). Incidence and Outcomes of Home Parenteral Nutrition in Patients With Crohn Disease in Olmsted County, Minnesota. Crohn’s Colitis 360.

[65] Cruz R., McGurgan J., Butera L., Poloyac K., Roberts M., Stein W., Minervini M., Jorgensen D., Humar A. (2020). Gastrointestinal Tract Reconstruction in Adults with Ultra-Short Bowel Syndrome: Surgical and Nutritional Outcomes. Surgery.

[66] Watanabe Y., Mizushima T., Fujino S., Ogino T., Miyoshi N., Takahashi H., Uemura M., Matsuda C., Yamamoto H., Doki Y. (2020). Long-term outcome of patients with Crohn’s disease on home parenteral nutrition. Nutrition.

[67] McKee R.F., Knight K., Leitch E.F., Stevens P. (2021). Changes in adult home parenteral nutrition practice over 25 years. Clin. Nutr. ESPEN.

[68] Noelting J., Gramlich L., Whittaker S., Armstrong D., Marliss E., Jurewitsch B., Raman M., Duerksen D.R., Stevenson D., Lou W. (2021). Survival of Patients with Short-Bowel Syndrome on Home Parenteral Nutrition: A Prospective Cohort Study. J. Parenter. Enter. Nutr..

[69] Gondolesi G.E., Ortega M.L., Doeyo M., Buncuga M., Perez C., Maurino E., Costa F., De Barrio S., Manzur A., Donnadio L. (2022). First registry of adult patients with chronic intestinal failure due to short bowel syndrome in Argentina: The RESTORE project. J. Parenter. Enter. Nutr..

[70] Thompson J.S., Rochling F.A., Lyden E., Merani S., Vargas L.M., Grant W.J., Langnas A.N., Mercer D.F. (2022). Cholecystectomy prior to short bowel syndrome does not alter nutritional prognosis. Am. J. Surg..

[71] Brito M., Padinha M., Carlos S., Oliveira C., Santos A.P., Nunes G., Santos C.A., Fonseca J. (2023). Long-Term Intestinal Failure and Home Parenteral Support: A Single Center Experience. GE Port. J. Gastroenterol..

[72] D’Eusebio C., Merlo F.D., Ossola M., Bioletto F., Ippolito M., Locatelli M., De Francesco A., Anro M., Romagnoli R., Strignano P. (2023). Mortality and parenteral nutrition weaning in patients with chronic intestinal failure on home parenteral nutrition: A 30-year retrospective cohort study. Nutrition.

[73] Santos M.D., Magalhaes V., Loureiro L., Pina P., Castro A., Aguiar P., Rocha A. (2024). Management of Short Bowel Syndrome With Chronic Intestinal Failure: A Single-Center Experience in Portugal. Cureus.

[74] Siddiqui M., Coughlin K., Koenen B., Al-Yaman W., Bestgen A., Regueiro M., Kirby D. (2024). Association between tunneled catheter placement and catheter-associated deep venous thrombosis in adults with inflammatory bowel disease receiving home parenteral nutrition: A retrospective cohort study. J. Parenter. Enter. Nutr..

[75] Salazar E., Clermont-Dejean N.M., Schwenger K.J.P., Noelting J., Lu Z., Lou W., Allard J.P. (2021). Patients With Severe Gastrointestinal Dysmotility Disorders Receiving Home Parenteral Nutrition Have Similar Survival As Those With Short-Bowel Syndrome: A Prospective Cohort Study. J. Parenter. Enter. Nutr..

[76] Stokes M., Irving M. (1989). Mortality in Patients on Home Parenteral-Nutrition. J. Parenter. Enter. Nutr..

[77] Kopczynska M., Carlson G., Teubner A., Abraham A., Taylor M., Burden S.T., Hvas C.L., Jepsen P., Lal S. (2022). Long-Term Outcomes in Patients with Intestinal Failure Due to Short Bowel Syndrome and Intestinal Fistula. Nutrients.

[78] McKee R.F., Knight K., Leitch E.F., Stevens P. (2022). The role of surgery in weaning patients from home parenteral support—A cohort study. Color. Dis..

[79] Borges V., da Silva M., Dias M., Gonzalez M., Waitzberg D. (2011). Long-term nutritional assessment of patients with severe short bowel syndrome managed with home enteral nutrition and oral intake. Nutr. Hosp..

[80] Mendes I., Vara-Luiz F., Palma C., Nunes G., Lima M.J., Oliveira C., Brito M., Santos A.P., Santos C.A., Fonseca J. (2024). Home Parenteral Support in Chronic Intestinal Failure-First Results from a Pioneer Portuguese Intestinal Failure Center. Nutrients.

[81] Pironi L., Boeykens K., Bozzetti F., Joly F., Klek S., Lal S., Lichota M., Mühlebach S., Van Gossum A., Wanten G. (2023). ESPEN practical guideline: Home parenteral nutrition. Clin. Nutr..

[82] Fuglsang K.A., Brandt C.F., Jeppesen P.B. (2022). Survival in patients initiating home parenteral support due to nonmalignant short bowel syndrome compared with background population. Clin. Nutr. ESPEN.

