# Supplementary material for: Clinical Outcomes on Home Parenteral Support for Patients with Benign Chronic Intestinal Failure: Systematic Review and Meta-Analysis
Source: Nutrients. 2026 Jul 1;18(13):2123. doi: 10.3390/nu18132123 (PMC13363228; doi:10.3390/nu18132123)

Table of Contents

Supplementary Table S1. The PRISMA 2020 checklist 2

Supplementary Table S2. Pooled estimates of the survival probabilities from 10 studies included in the meta-analysis for patients with benign intestinal failure on home parenteral support. 4

Supplementary Figure S1. Search strategies for all databases 5

Supplementary Figure S2. The time period for the included studies in years from 1969 to 2023 14

Supplementary Figure S3. Sub-group analysis of the overall mortality 15

Supplementary Figure S4. Previously reported associations of all-cause mortality from studies included in the systematic review. 18

Supplementary Figure S5. Sub-group analysis of the regaining nutritional autonomy 19

Supplementary Figure S6. Previously reported associations of regaining nutritional autonomy from studies included in the systematic review. 22

Supplementary Figure S7. Risk of Bias Chart for studies included in the systematic review. 23

Section and Topic	Item #	Checklist item	Location where item is reported
**TITLE**			
Title	1	Identify the report as a systematic review.	1
**ABSTRACT**			
Abstract	2	See the PRISMA 2020 for Abstracts checklist.	1
**INTRODUCTION**			
Rationale	3	Describe the rationale for the review in the context of existing knowledge.	2-3
Objectives	4	Provide an explicit statement of the objective(s) or question(s) the review addresses.	3
**METHODS**			
Eligibility criteria	5	Specify the inclusion and exclusion criteria for the review and how studies were grouped for the syntheses.	3
Information sources	6	Specify all databases, registers, websites, organisations, reference lists and other sources searched or consulted to identify studies. Specify the date when each source was last searched or consulted.	3
Search strategy	7	Present the full search strategies for all databases, registers and websites, including any filters and limits used.	3
Selection process	8	Specify the methods used to decide whether a study met the inclusion criteria of the review, including how many reviewers screened each record and each report retrieved, whether they worked independently, and if applicable, details of automation tools used in the process.	4
Data collection process	9	Specify the methods used to collect data from reports, including how many reviewers collected data from each report, whether they worked independently, any processes for obtaining or confirming data from study investigators, and if applicable, details of automation tools used in the process.	4
Data items	10a	List and define all outcomes for which data were sought. Specify whether all results that were compatible with each outcome domain in each study were sought (e.g. for all measures, time points, analyses), and if not, the methods used to decide which results to collect.	4
	10b	List and define all other variables for which data were sought (e.g. participant and intervention characteristics, funding sources). Describe any assumptions made about any missing or unclear information.	4
Study risk of bias assessment	11	Specify the methods used to assess risk of bias in the included studies, including details of the tool(s) used, how many reviewers assessed each study and whether they worked independently, and if applicable, details of automation tools used in the process.	4
Effect measures	12	Specify for each outcome the effect measure(s) (e.g. risk ratio, mean difference) used in the synthesis or presentation of results.	4
Synthesis methods	13a	Describe the processes used to decide which studies were eligible for each synthesis (e.g. tabulating the study intervention characteristics and comparing against the planned groups for each synthesis (item #5)).	4-5
	13b	Describe any methods required to prepare the data for presentation or synthesis, such as handling of missing summary statistics, or data conversions.	4-5
	13c	Describe any methods used to tabulate or visually display results of individual studies and syntheses.	4-5
	13d	Describe any methods used to synthesize results and provide a rationale for the choice(s). If meta-analysis was performed, describe the model(s), method(s) to identify the presence and extent of statistical heterogeneity, and software package(s) used.	4-5
	13e	Describe any methods used to explore possible causes of heterogeneity among study results (e.g. subgroup analysis, meta-regression).	4-5
	13f	Describe any sensitivity analyses conducted to assess robustness of the synthesized results.	4-5
Reporting bias assessment	14	Describe any methods used to assess risk of bias due to missing results in a synthesis (arising from reporting biases).	4
Certainty	15	Describe any methods used to assess certainty (or confidence) in the body of evidence for an outcome.	4-5

Section and Topic	Item #	Checklist item	Location where item is reported
assessment			
**RESULTS**			
Study selection	16a	Describe the results of the search and selection process, from the number of records identified in the search to the number of studies included in the review, ideally using a flow diagram.	5
	16b	Cite studies that might appear to meet the inclusion criteria, but which were excluded, and explain why they were excluded.	5
Study characteristics	17	Cite each included study and present its characteristics.	6-11
Risk of bias in studies	18	Present assessments of risk of bias for each included study.	17
Results of individual studies	19	For all outcomes, present, for each study: (a) summary statistics for each group (where appropriate) and (b) an effect estimate and its precision (e.g. confidence/credible interval), ideally using structured tables or plots.	12-17
Results of syntheses	20a	For each synthesis, briefly summarise the characteristics and risk of bias among contributing studies.	12-17
	20b	Present results of all statistical syntheses conducted. If meta-analysis was done, present for each the summary estimate and its precision (e.g. confidence/credible interval) and measures of statistical heterogeneity. If comparing groups, describe the direction of the effect.	12-17
	20c	Present results of all investigations of possible causes of heterogeneity among study results.	12-17
	20d	Present results of all sensitivity analyses conducted to assess the robustness of the synthesized results.	12-17
Reporting biases	21	Present assessments of risk of bias due to missing results (arising from reporting biases) for each synthesis assessed.	17
Certainty of evidence	22	Present assessments of certainty (or confidence) in the body of evidence for each outcome assessed.	12-17
**DISCUSSION**			
Discussion	23a	Provide a general interpretation of the results in the context of other evidence.	17-20
	23b	Discuss any limitations of the evidence included in the review.	19-20
	23c	Discuss any limitations of the review processes used.	19-20
	23d	Discuss implications of the results for practice, policy, and future research.	17-20
**OTHER INFORMATION**			
Registration and protocol	24a	Provide registration information for the review, including register name and registration number, or state that the review was not registered.	3
	24b	Indicate where the review protocol can be accessed, or state that a protocol was not prepared.	3
	24c	Describe and explain any amendments to information provided at registration or in the protocol.	NA
Support	25	Describe sources of financial or non-financial support for the review, and the role of the funders or sponsors in the review.	NA
Competing interests	26	Declare any competing interests of review authors.	20
Availability of data, code and other materials	27	Report which of the following are publicly available and where they can be found: template data collection forms; data extracted from included studies; data used for all analyses; analytic code; any other materials used in the review.	20

Supplementary Table S1. The PRISMA 2020 checklist

Follow-up (years)	Summary survival (%)	95% CI
1	92.56%	90.31% to 94.86%
2	85.19%	82.99% to 87.44%
3	78.30%	74.63% to 82.15%
4	71.71%	65.52% to 78.47%
5	68.71%	62.64% to 75.37%
6	66.47%	59.93% to 73.73%
7	62.85%	56.50% to 69.92%
8	58.45%	52.39% to 65.22%
9	55.79%	49.85% to 62.44%
10	52.46%	46.76% to 58.86%

Supplementary Table S2. Pooled estimates of the survival probabilities from 10 studies included in the meta-analysis for patients with benign intestinal failure on home parenteral support.

Supplementary Figure S1. Search strategies for all databases

Medline(R) 1946 to February 03, 2025 (Ovid Platform)

1	intestinal failure.mp.
2	short bowel syndrome.mp.
3	short gut syndrome.mp.
4	short gut.mp.
5	exp *parenteral nutrition/
6	exp Parenteral Nutrition Solutions/
7	parenteral nutrition*.mp.
8	total parenteral nutrition.mp.
9	home parenteral nutrition.mp.
10	hyperalimentation.mp.
11	intravenous feeding.mp.
12	(parenteral* adj3 (nutri* or hydration* or feed* or fed* or treatment* or manag* or method* or car* or support* or diet*)).mp.
13	(nutrition adj5 (venous line* or central line* or Hickman line*)).mp.
14	((parenteral or intravenous) adj5 (nutrition or feeding)).mp.
15	(parenteral$ adj2 (fed or feed$)).ti,ab.
16	Catheterization/
17	Catheterization, Central Venous/
18	Catheters/
19	Catheters, Indwelling/
20	Central Venous Catheters/
21	(catheter* and (central or in-dwelling* or indwelling*)).mp.
22	CVC*.mp.
23	survival rate/
24	survival analysis/
25	survivor/
26	mortality/
27	mortality rate/
28	death/
29	survival/
30	survival factor/
31	surviv*.mp.
32	mortality.mp.
33	death*.mp.
34	dead*.mp.

35 fatal*.mp.

36 outcome.mp.

37 natural history.mp.

38 IFALD.mp.

39 Intestinal Failure Associated Liver Disease.mp.

40 PNALD.mp.

41 parenteral nutrition-associated liver disease.mp.

42 PNAC.mp.

43 Parenteral nutrition associated cholestasis.mp.

44 PNALI.mp.

45 Parenteral nutrition Associated Liver Injury.mp.

46 Catheter-Related Infections.mp.

47 CRBSI.mp.

48 catheter related bloodstream infections.mp.

49 CLABSI.mp.

50 central line associated bloodstream infections.mp.

51 bloodstream infection.mp.

52 Bacterial Infections.mp.

53 Sepsis.mp.

54 septicemia.mp.

55 septicaemia.mp.

56 Cross Infection.mp.

57 exp Infection/

58 infect*.mp.

59 Bacteremia*.mp.

60 fungemia*.mp.

61 fungus.mp.

62 candidemia*.mp.

63 candidiasis.mp.

64 candida.mp.

65 catheter occlusion.mp.

66 catheter thrombosis.mp.

67 exp Thrombosis/

68 thromboses.mp.

69 thromboembolism.mp.

70 Thrombus.mp.

71 renal impairment.mp.

72 renal function.mp.

73 renal dysfunction.mp.

74 renal failure.mp.

75 chronic kidney disease.mp.

76 chronic kidney failure.mp.

77 end stage renal disease.mp.

78 gallbladder.mp.

79 cholecystitis.mp.

80 gallstone.mp.

81 calculi.mp.

82 cholelithiasis.mp.

83 cholecystolithiasis.mp.

84 choledocholithiasis.mp.

85 bile duct stone.mp.

86 cbd stone.mp.

87 biliary disease.mp.

88 biliary tract disease.mp.

89 gallbladder inflammation.mp.

90 bone.mp.

91 bone health.mp.

92 metabolic bone disease.mp.

93 osteoporosis.mp.

94 osteopenia.mp.

95 intestinal transplant.mp.

96 small bowel transplant.mp.

97 (combined liver and small bowel transplant).mp.

98 multivisceral transplant.mp.

99 exp adolescent/

100 exp Child/

101 adolescent*.mp.

102 child*.mp.

103 schoolchild*.mp.

104 infant*.mp.

105 girl*.mp.

106 boy*.mp.

107 teen.mp.

108 teens.mp.

109 teenager*.mp.

110 youth*.mp.

111 pediatr*.mp.

112 paediatr*.mp.

113 puber*.mp.

114 exp animals/ not humans.sh.

115 1 or 2 or 3 or 4

116 5 or 6 or 7 or 8 or 9 or 10 or 11 or 12 or 13 or 14 or 15

117 16 or 17 or 18 or 19 or 20 or 21 or 22 or 23 or 24 or 25 or 26 or 27 or 28 or 29 or 30 or 31 or 32 or 33 or 34 or 35 or 36 or 37 or 38 or 39 or 40 or 41 or 42 or 43 or 44 or 45 or 46 or 47 or 48 or 49 or 50 or 51 or 52 or 53 or 54 or 55 or 56 or 57 or 58 or 59 or 60 or 61 or 62 or 63 or 64 or 65 or 66 or 67 or 68 or 69 or 70 or 71 or 72 or 73 or 74 or 75 or 76 or 77 or 78 or 79 or 80 or 81 or 82 or 83 or 84 or 85 or 86 or 87 or 88 or 89 or 90 or 91 or 92 or 93 or 94 or 95 or 96 or 97 or 98

118 99 or 100 or 101 or 102 or 103 or 104 or 105 or 106 or 107 or 108 or 109 or 110 or 111 or 112 or 113 or 114

119 (115 and 116 and 117) not 118

120 (115 and (116 or 117)) not 118

Embase 1974 to 2025 February 03 (Ovid platform)

1	intestinal failure.mp.
2	short bowel syndrome.mp.
3	short gut syndrome.mp.
4	short gut.mp.
5	exp *parenteral nutrition/
6	exp Parenteral Nutrition Solutions/
7	parenteral nutrition*.mp.
8	total parenteral nutrition.mp.
9	home parenteral nutrition.mp.
10	hyperalimentation.mp.
11	intravenous feeding.mp.
12	(parenteral* adj3 (nutri* or hydration* or feed* or fed* or treatment* or manag* or method* or car* or support* or diet*)).mp.
13	(nutrition adj5 (venous line* or central line* or Hickman line*)).mp.
14	((parenteral or intravenous) adj5 (nutrition or feeding)).mp.
15	(parenteral$ adj2 (fed or feed$)).ti,ab.
16	Catheterization/
17	Catheterization, Central Venous/
18	Catheters/
19	Catheters, Indwelling/
20	Central Venous Catheters/
21	(catheter* and (central or in-dwelling* or indwelling*)).mp.
22	CVC*.mp.
23	survival rate/
24	survival analysis/
25	survivor/
26	mortality/
27	mortality rate/
28	death/
29	survival/
30	survival factor/
31	surviv*.mp.
32	mortality.mp.
33	death*.mp.
34	dead*.mp.
35	fatal*.mp.

36 outcome.mp.

37 natural history.mp.

38 IFALD.mp.

39 Intestinal Failure Associated Liver Disease.mp.

40 PNALD.mp.

41 parenteral nutrition-associated liver disease.mp.

42 PNAC.mp.

43 Parenteral nutrition associated cholestasis.mp.

44 PNALI.mp.

45 Parenteral nutrition Associated Liver Injury.mp.

46 Catheter-Related Infections.mp.

47 CRBSI.mp.

48 catheter related bloodstream infections.mp.

49 CLABSI.mp.

50 central line associated bloodstream infections.mp.

51 bloodstream infection.mp.

52 Bacterial Infections.mp.

53 Sepsis.mp.

54 septicemia.mp.

55 septicaemia.mp.

56 Cross Infection.mp.

57 exp Infection/

58 infect*.mp.

59 Bacteremia*.mp.

60 fungemia*.mp.

61 fungus.mp.

62 candidemia*.mp.

63 candidiasis.mp.

64 candida.mp.

65 catheter occlusion.mp.

66 catheter thrombosis.mp.

67 exp Thrombosis/

68 thromboses.mp.

69 thromboembolism.mp.

70 Thrombus.mp.

71 renal impairment.mp.

72 renal function.mp.

73 renal dysfunction.mp.

74 renal failure.mp.

75 chronic kidney disease.mp.

76 chronic kidney failure.mp.

77 end stage renal disease.mp.

78 gallbladder.mp.

79 cholecystitis.mp.

80 gallstone.mp.

81 calculi.mp.

82 cholelithiasis.mp.

83 cholecystolithiasis.mp.

84 choledocholithiasis.mp.

85 bile duct stone.mp.

86 cbd stone.mp.

87 biliary disease.mp.

88 biliary tract disease.mp.

89 gallbladder inflammation.mp.

90 bone.mp.

91 bone health.mp.

92 metabolic bone disease.mp.

93 osteoporosis.mp.

94 osteopenia.mp.

95 intestinal transplant.mp.

96 small bowel transplant.mp.

97 (combined liver and small bowel transplant).mp.

98 multivisceral transplant.mp.

99 exp adolescent/

100 exp Child/

101 adolescent*.mp.

102 child*.mp.

103 schoolchild*.mp.

104 infant*.mp.

105 girl*.mp.

106 boy*.mp.

107 teen.mp.

108 teens.mp.

109 teenager*.mp.

110 youth*.mp.

111 pediatr*.mp.

112 paediatr*.mp.

113 puber*.mp.

114 exp animals/ not humans.sh.

115 1 or 2 or 3 or 4

116 5 or 6 or 7 or 8 or 9 or 10 or 11 or 12 or 13 or 14 or 15

117 16 or 17 or 18 or 19 or 20 or 21 or 22 or 23 or 24 or 25 or 26 or 27 or 28 or 29 or 30 or 31 or 32 or 33 or 34 or 35 or 36 or 37 or 38 or 39 or 40 or 41 or 42 or 43 or 44 or 45 or 46 or 47 or 48 or 49 or 50 or 51 or 52 or 53 or 54 or 55 or 56 or 57 or 58 or 59 or 60 or 61 or 62 or 63 or 64 or 65 or 66 or 67 or 68 or 69 or 70 or 71 or 72 or 73 or 74 or 75 or 76 or 77 or 78 or 79 or 80 or 81 or 82 or 83 or 84 or 85 or 86 or 87 or 88 or 89 or 90 or 91 or 92 or 93 or 94 or 95 or 96 or 97 or 98

118 99 or 100 or 101 or 102 or 103 or 104 or 105 or 106 or 107 or 108 or 109 or 110 or 111 or 112 or 113 or 114

119 (115 and 116 and 117) not 118

120 (115 and (116 or 117)) not 118

Web of Science (Clarivate platform)

TS=("intestinal failure" OR "short bowel syndrome" OR "short gut syndrome" OR "short gut") AND (TS=("parenteral nutrition*" OR "total parenteral nutrition" OR "home parenteral nutrition" OR "hyperalimentation" OR "intravenous feeding") OR TS=("IFALD" OR "Intestinal Failure Associated Liver Disease" OR "PNALD" OR "parenteral nutrition-associated liver disease" OR "PNAC" OR "Parenteral nutrition associated cholestasis" OR "PNALI" OR "Parenteral nutrition Associated Liver Injury" OR "Catheterization" OR "Catheterization, Central Venous" OR "Catheters" OR "Catheters, Indwelling" OR "Central Venous Catheters" OR "CVC*" OR "Catheter-Related Infections" OR "CRBSI" OR "catheter related bloodstream infections" OR "CLABSI" OR "central line associated bloodstream infections" OR "bloodstream infection" OR "Bacterial Infections" OR "Sepsis" OR "septicemia" OR "septicaemia" OR "Cross Infection" OR "infect*" OR "Bacteremia*" OR "fungemia*" OR "fungus" OR "candidemia*" OR "candidiasis" OR "candida" OR "catheter occlusion" OR "catheter thrombosis" OR "Thrombosis" OR "thromboses" OR "thromboembolism" OR "Thrombus" OR "renal impairment" OR "renal function" OR "renal dysfunction" OR "renal failure" OR "chronic kidney disease" OR "chronic kidney failure" OR "end stage renal disease" OR "gallbladder" OR "cholecystitis " OR "gallstone" OR "calculi" OR "cholelithiasis" OR "cholecystolithiasis" OR "choledocholithiasis " OR "bile duct stone" OR "cbd stone" OR "biliary disease" OR "biliary tract disease" OR "gallbladder inflammation" OR "bone" OR "bone health" OR "metabolic bone disease" OR "osteoporosis" OR "osteopenia" OR "intestinal transplant." OR "small bowel transplant." OR "combined liver and small bowel transplant." OR "multivisceral transplant." OR "survival rate " OR "survival analysis" OR "survivor" OR "mortality " OR "mortality rate" OR "death" OR "survival" OR "survival factor" OR "surviv*" OR "mortality" OR "death*" OR "dead*" OR "fatal*" OR "outcome" OR "natural history")) NOT TS=("child*" OR "schoolchild*" OR "infant*" OR "girl*" OR "boy*" OR "teen" OR "teens" OR "teenager*" OR "youth*" OR "pediatr*" OR "paediatr*" OR "puber*" OR "animal*" OR "not humans")

Supplementary Figure S2. The time period for the included studies in years from 1969 to 2023. Of note, two studies are not presented in the graph due to lack of data [25] or cross-sectional design [35].

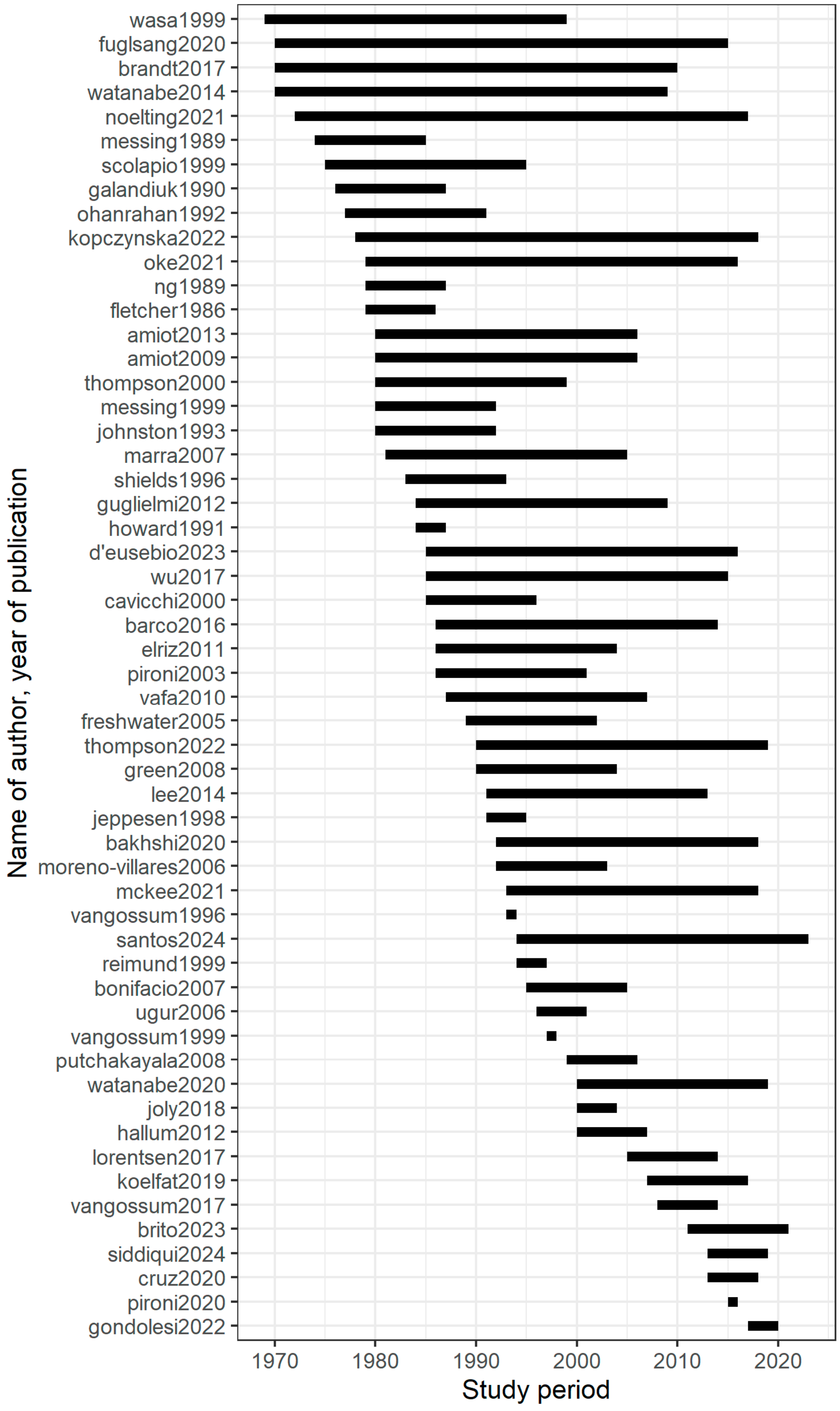

Supplementary Figure S3. Sub-group analysis of the overall mortality, stratified by (**A**) centre size, (**B**) follow-up type, (**C**) reported risk of bias in study.

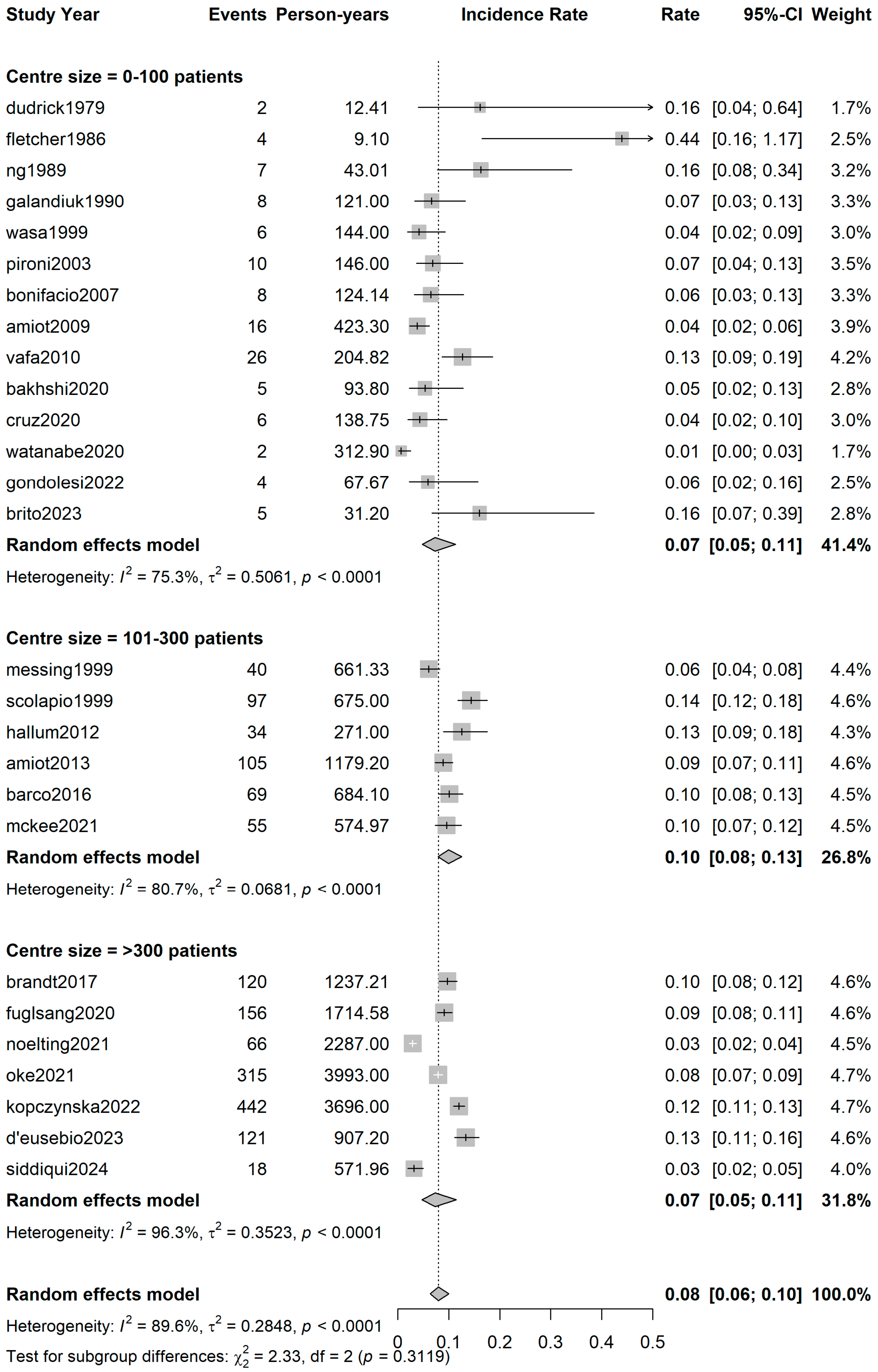

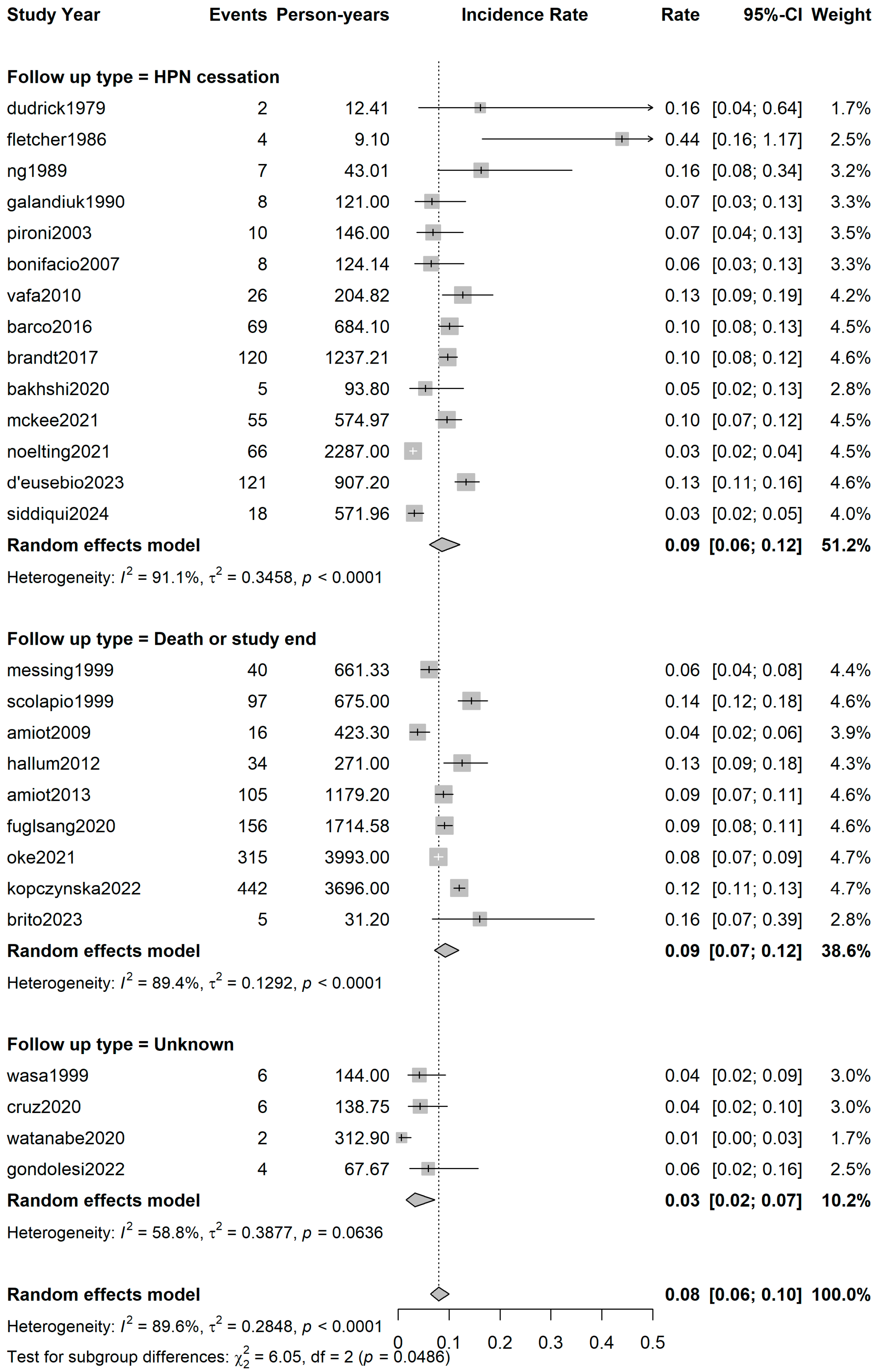
Study Year
Events
Person-years
Incidence Rate
Rate
95%-CI
Weight
Follow up type = HPN cessation
dudrick1979 2 12.41 0.16 [0.04; 0.64] 1.7%
fletcher1986 4 9.10 0.44 [0.16; 1.17] 2.5%
ng1989 7 43.01 0.16 [0.08; 0.34] 3.2%
galandiuk1990 8 121.00 0.07 [0.03; 0.13] 3.3%
pironi2003 10 146.00 0.07 [0.04; 0.13] 3.5%
bonifacio2007 8 124.14 0.06 [0.03; 0.13] 3.3%
vafa2010 26 204.82 0.13 [0.09; 0.19] 4.2%
barco2016 69 684.10 0.10 [0.08; 0.13] 4.5%
brandt2017 120 1237.21 0.10 [0.08; 0.12] 4.6%
bakhshi2020 5 93.80 0.05 [0.02; 0.13] 2.8%
mckee2021 55 574.97 0.10 [0.07; 0.12] 4.5%
noelting2021 66 2287.00 0.03 [0.02; 0.04] 4.5%
d'eusebio2023 121 907.20 0.13 [0.11; 0.16] 4.6%
siddiqui2024 18 571.96 0.03 [0.02; 0.05] 4.0%
Random effects model 0.09 [0.06; 0.12] 51.2%
Heterogeneity: I^2 = 91.1%, τ^2 = 0.3458, p < 0.0001
Follow up type = Death or study end
messing1999 40 661.33 0.06 [0.04; 0.08] 4.4%
scolapio1999 97 675.00 0.14 [0.12; 0.18] 4.6%
amiot2009 16 423.30 0.04 [0.02; 0.06] 3.9%
hallum2012 34 271.00 0.13 [0.09; 0.18] 4.3%
amiot2013 105 1179.20 0.09 [0.07; 0.11] 4.6%
fuglsang2020 156 1714.58 0.09 [0.08; 0.11] 4.6%
oke2021 315 3993.00 0.08 [0.07; 0.09] 4.7%
kopczynska2022 442 3696.00 0.12 [0.11; 0.13] 4.7%
brito2023 5 31.20 0.16 [0.07; 0.39] 2.8%
Random effects model 0.09 [0.07; 0.12] 38.6%
Heterogeneity: I^2 = 89.4%, τ^2 = 0.1292, p < 0.0001
Follow up type = Unknown
wasa1999 6 144.00 0.04 [0.02; 0.09] 3.0%
cruz2020 6 138.75 0.04 [0.02; 0.10] 3.0%
watanabe2020 2 312.90 0.01 [0.00; 0.03] 1.7%
gondolesi2022 4 67.67 0.06 [0.02; 0.16] 2.5%
Random effects model 0.03 [0.02; 0.07] 10.2%
Heterogeneity: I^2 = 58.8%, τ^2 = 0.3877, p = 0.0636
Random effects model 0.08 [0.06; 0.10] 100.0%
Heterogeneity: I^2 = 89.6%, τ^2 = 0.2848, p < 0.0001
Test for subgroup differences: χ^2_2 = 6.05, df = 2 (p = 0.0486)
0
0.1
0.2
0.3
0.4
0.5

Study Year	Events	Person-years	Rate	95%-CI	Weight
**Risk of bias = Some concern**					
dudrick1979	2	12.41	0.16	[0.04; 0.64]	1.7%
fletcher1986	4	9.10	0.44	[0.16; 1.17]	2.5%
ng1989	7	43.01	0.16	[0.08; 0.34]	3.2%
messing1999	40	661.33	0.06	[0.04; 0.08]	4.4%
wasa1999	6	144.00	0.04	[0.02; 0.09]	3.0%
pironi2003	10	146.00	0.07	[0.04; 0.13]	3.5%
amiot2009	16	423.30	0.04	[0.02; 0.06]	3.9%
hallum2012	34	271.00	0.13	[0.09; 0.18]	4.3%
amiot2013	105	1179.20	0.09	[0.07; 0.11]	4.6%
brandt2017	120	1237.21	0.10	[0.08; 0.12]	4.6%
bakhshi2020	5	93.80	0.05	[0.02; 0.13]	2.8%
cruz2020	6	138.75	0.04	[0.02; 0.10]	3.0%
fuglsang2020	156	1714.58	0.09	[0.08; 0.11]	4.6%
noelting2021	66	2287.00	0.03	[0.02; 0.04]	4.5%
oke2021	315	3993.00	0.08	[0.07; 0.09]	4.7%
gondolesi2022	4	67.67	0.06	[0.02; 0.16]	2.5%
siddiqui2024	18	571.96	0.03	[0.02; 0.05]	4.0%
**Random effects model**			**0.07**	**[0.05; 0.10]**	**61.8%**
Heterogeneity: $I^2 = 87.9\%$, $\tau^2 = 0.2932$, $p < 0.0001$					
**Risk of bias = Low risk**					
galandiuk1990	8	121.00	0.07	[0.03; 0.13]	3.3%
scolapio1999	97	675.00	0.14	[0.12; 0.18]	4.6%
bonifacio2007	8	124.14	0.06	[0.03; 0.13]	3.3%
barco2016	69	684.10	0.10	[0.08; 0.13]	4.5%
watanabe2020	2	312.90	0.01	[0.00; 0.03]	1.7%
mckee2021	55	574.97	0.10	[0.07; 0.12]	4.5%
kopczynska2022	442	3696.00	0.12	[0.11; 0.13]	4.7%
brito2023	5	31.20	0.16	[0.07; 0.39]	2.8%
d'eusebio2023	121	907.20	0.13	[0.11; 0.16]	4.6%
**Random effects model**			**0.10**	**[0.08; 0.13]**	**34.0%**
Heterogeneity: $I^2 = 75.6\%$, $\tau^2 = 0.0922$, $p < 0.0001$					
**Risk of bias = h**					
vafa2010	26	204.82	0.13	[0.09; 0.19]	4.2%
**Random effects model**			**0.08**	**[0.06; 0.10]**	**100.0%**

Heterogeneity: $I^2 = 89.6\%$, $\tau^2 = 0.2848$, $p < 0.0001$

Test for subgroup differences: $\chi^2_2 = 6.11$, df = 2 ($p = 0.0472$)

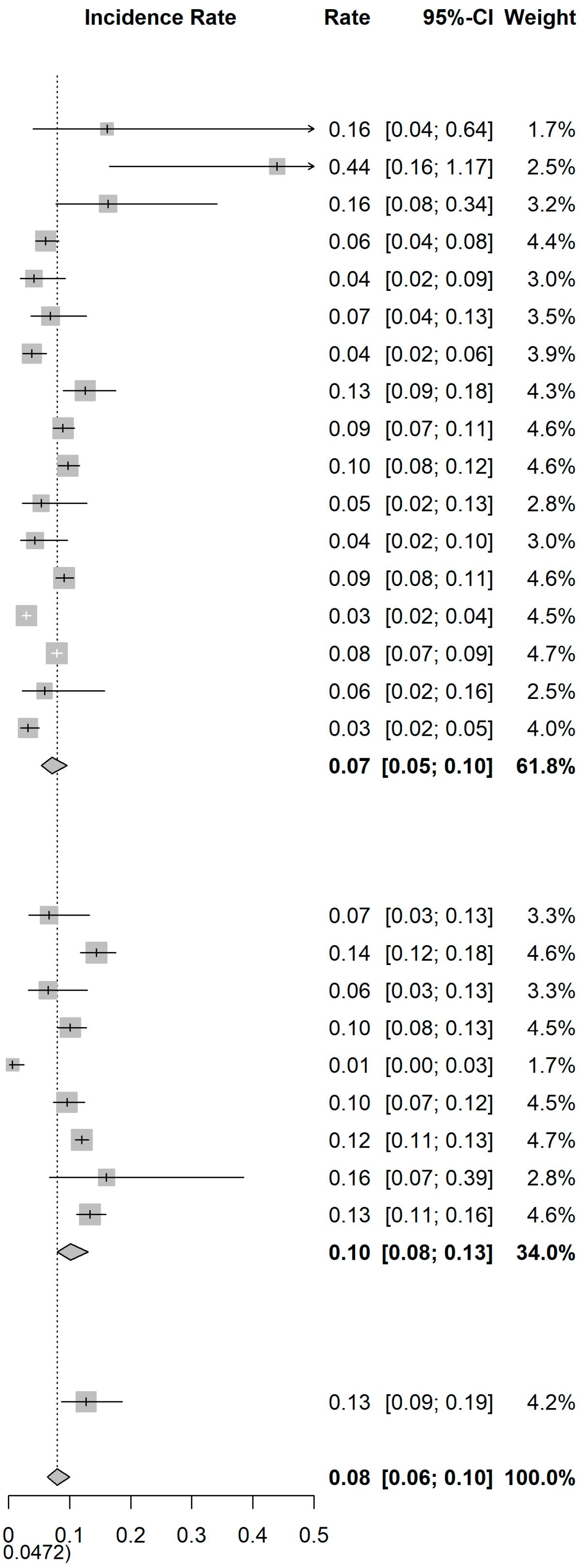

Supplementary Figure S4. Previously reported associations of all-cause mortality from studies included in the systematic review.

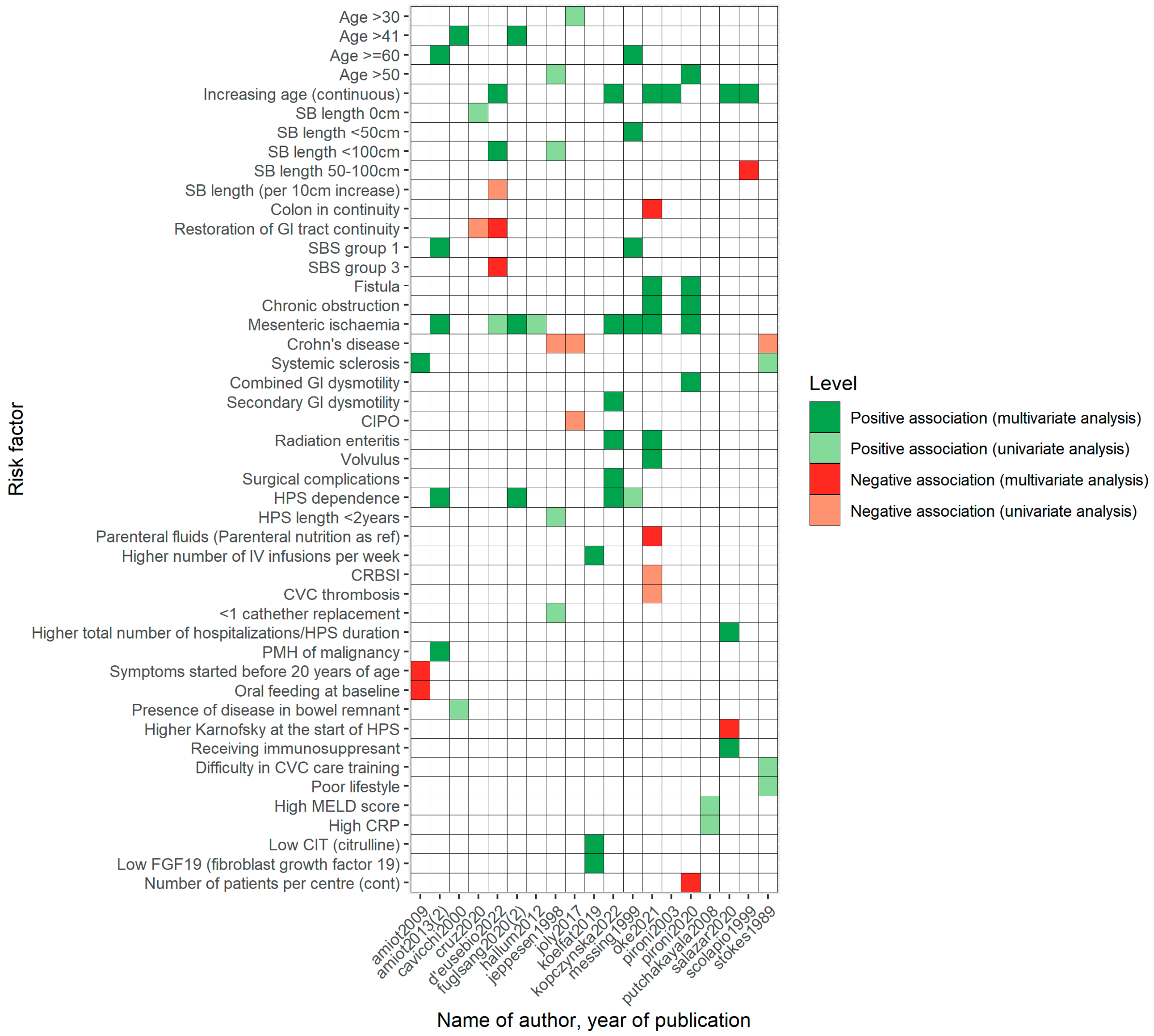

Supplementary Figure S5. Sub-group analysis of the regaining nutritional autonomy, stratified by (**A**) centre size, (**B**) analysis type, (**C**) reported risk of bias in study.

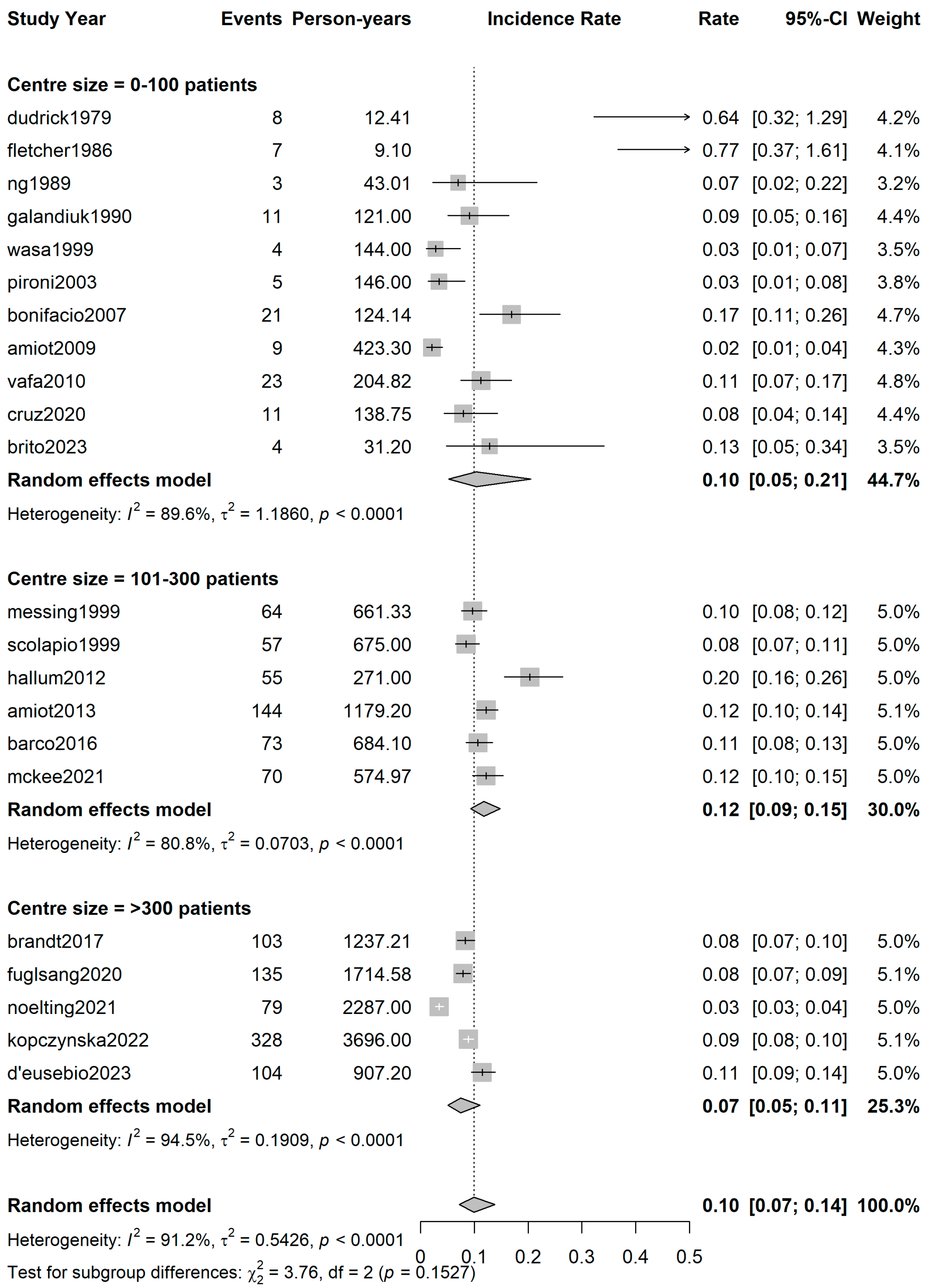

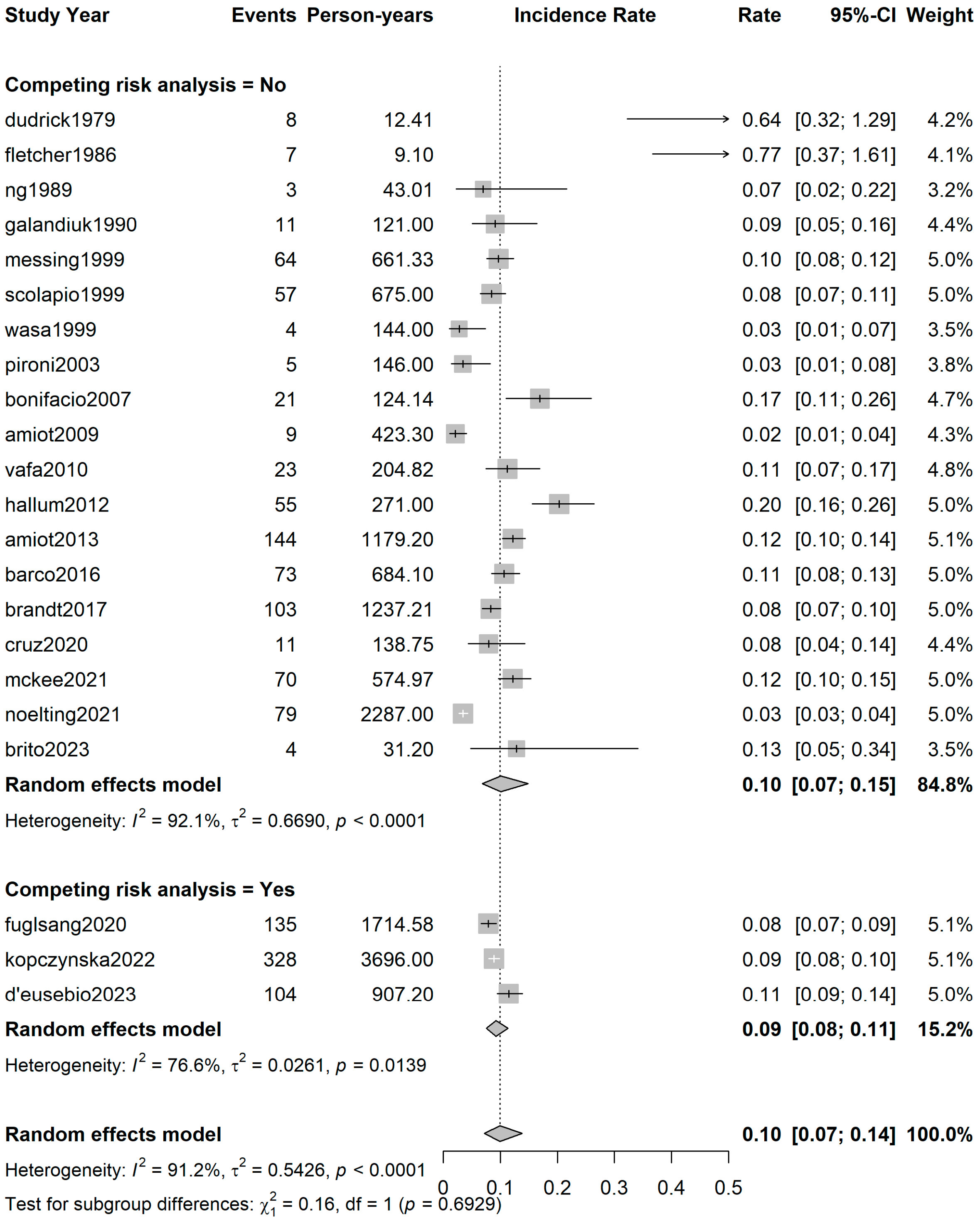
Study Year Events Person-years Incidence Rate Rate 95%-CI Weight
Competing risk analysis = No
dudrick1979 8 12.41 0.64 [0.32; 1.29] 4.2%
fletcher1986 7 9.10 0.77 [0.37; 1.61] 4.1%
ng1989 3 43.01 0.07 [0.02; 0.22] 3.2%
galandiuk1990 11 121.00 0.09 [0.05; 0.16] 4.4%
messing1999 64 661.33 0.10 [0.08; 0.12] 5.0%
scolapio1999 57 675.00 0.08 [0.07; 0.11] 5.0%
wasa1999 4 144.00 0.03 [0.01; 0.07] 3.5%
pironi2003 5 146.00 0.03 [0.01; 0.08] 3.8%
bonifacio2007 21 124.14 0.17 [0.11; 0.26] 4.7%
amiot2009 9 423.30 0.02 [0.01; 0.04] 4.3%
vafa2010 23 204.82 0.11 [0.07; 0.17] 4.8%
hallum2012 55 271.00 0.20 [0.16; 0.26] 5.0%
amiot2013 144 1179.20 0.12 [0.10; 0.14] 5.1%
barco2016 73 684.10 0.11 [0.08; 0.13] 5.0%
brandt2017 103 1237.21 0.08 [0.07; 0.10] 5.0%
cruz2020 11 138.75 0.08 [0.04; 0.14] 4.4%
mckee2021 70 574.97 0.12 [0.10; 0.15] 5.0%
noelting2021 79 2287.00 0.03 [0.03; 0.04] 5.0%
brito2023 4 31.20 0.13 [0.05; 0.34] 3.5%
Random effects model 0.10 [0.07; 0.15] 84.8%
Heterogeneity: I^2 = 92.1%, τ^2 = 0.6690, p < 0.0001
Competing risk analysis = Yes
fuglsang2020 135 1714.58 0.08 [0.07; 0.09] 5.1%
kopczynska2022 328 3696.00 0.09 [0.08; 0.10] 5.1%
d'eusebio2023 104 907.20 0.11 [0.09; 0.14] 5.0%
Random effects model 0.09 [0.08; 0.11] 15.2%
Heterogeneity: I^2 = 76.6%, τ^2 = 0.0261, p = 0.0139
Random effects model 0.10 [0.07; 0.14] 100.0%
Heterogeneity: I^2 = 91.2%, τ^2 = 0.5426, p < 0.0001
Test for subgroup differences: χ^2_1 = 0.16, df = 1 (p = 0.6929)
0 0.1 0.2 0.3 0.4 0.5

Study Year	Events	Person-years	Rate	95%-CI	Weight
**Risk of bias = Some concern**					
dudrick1979	8	12.41	0.64	[0.32; 1.29]	4.2%
fletcher1986	7	9.10	0.77	[0.37; 1.61]	4.1%
ng1989	3	43.01	0.07	[0.02; 0.22]	3.2%
messing1999	64	661.33	0.10	[0.08; 0.12]	5.0%
wasa1999	4	144.00	0.03	[0.01; 0.07]	3.5%
pironi2003	5	146.00	0.03	[0.01; 0.08]	3.8%
amiot2009	9	423.30	0.02	[0.01; 0.04]	4.3%
hallum2012	55	271.00	0.20	[0.16; 0.26]	5.0%
amiot2013	144	1179.20	0.12	[0.10; 0.14]	5.1%
brandt2017	103	1237.21	0.08	[0.07; 0.10]	5.0%
cruz2020	11	138.75	0.08	[0.04; 0.14]	4.4%
fuglsang2020	135	1714.58	0.08	[0.07; 0.09]	5.1%
noelting2021	79	2287.00	0.03	[0.03; 0.04]	5.0%
**Random effects model**			**0.09**	**[0.05; 0.17]**	**57.5%**
Heterogeneity: $I^2 = 94.5\%$, $\tau^2 = 1.0558$, $p < 0.0001$					
**Risk of bias = Low risk**					
galandiuk1990	11	121.00	0.09	[0.05; 0.16]	4.4%
scolapio1999	57	675.00	0.08	[0.07; 0.11]	5.0%
bonifacio2007	21	124.14	0.17	[0.11; 0.26]	4.7%
barco2016	73	684.10	0.11	[0.08; 0.13]	5.0%
mckee2021	70	574.97	0.12	[0.10; 0.15]	5.0%
kopczynska2022	328	3696.00	0.09	[0.08; 0.10]	5.1%
brito2023	4	31.20	0.13	[0.05; 0.34]	3.5%
d'eusebio2023	104	907.20	0.11	[0.09; 0.14]	5.0%
**Random effects model**			**0.11**	**[0.09; 0.12]**	**37.8%**
Heterogeneity: $I^2 = 59.7\%$, $\tau^2 = 0.0220$, $p = 0.0151$					
**Risk of bias = High risk**					
vafa2010	23	204.82	0.11	[0.07; 0.17]	4.8%
**Random effects model**			**0.10**	**[0.07; 0.14]**	**100.0%**

Heterogeneity: $I^2 = 91.2\%$, $\tau^2 = 0.5426$, $p < 0.0001$

Test for subgroup differences: $\chi^2_2 = 0.30$, df = 2 ($p = 0.8627$)

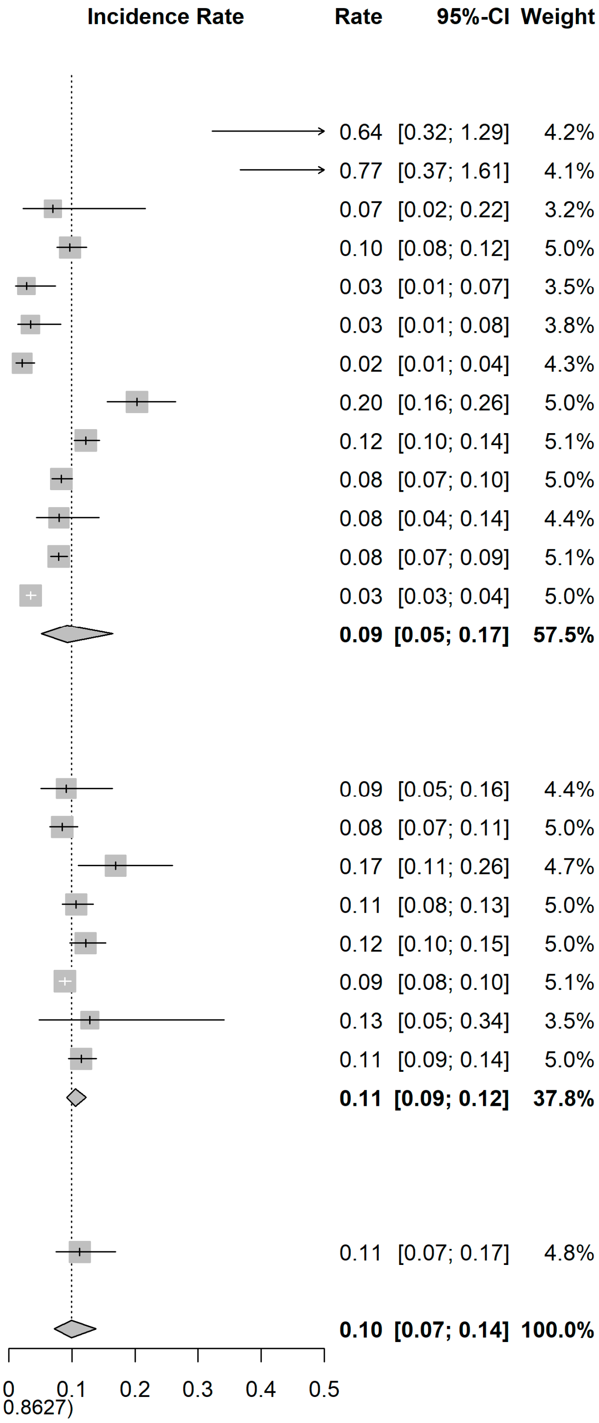

Supplementary Figure S6. Previously reported associations of regaining nutritional autonomy from studies included in the systematic review.

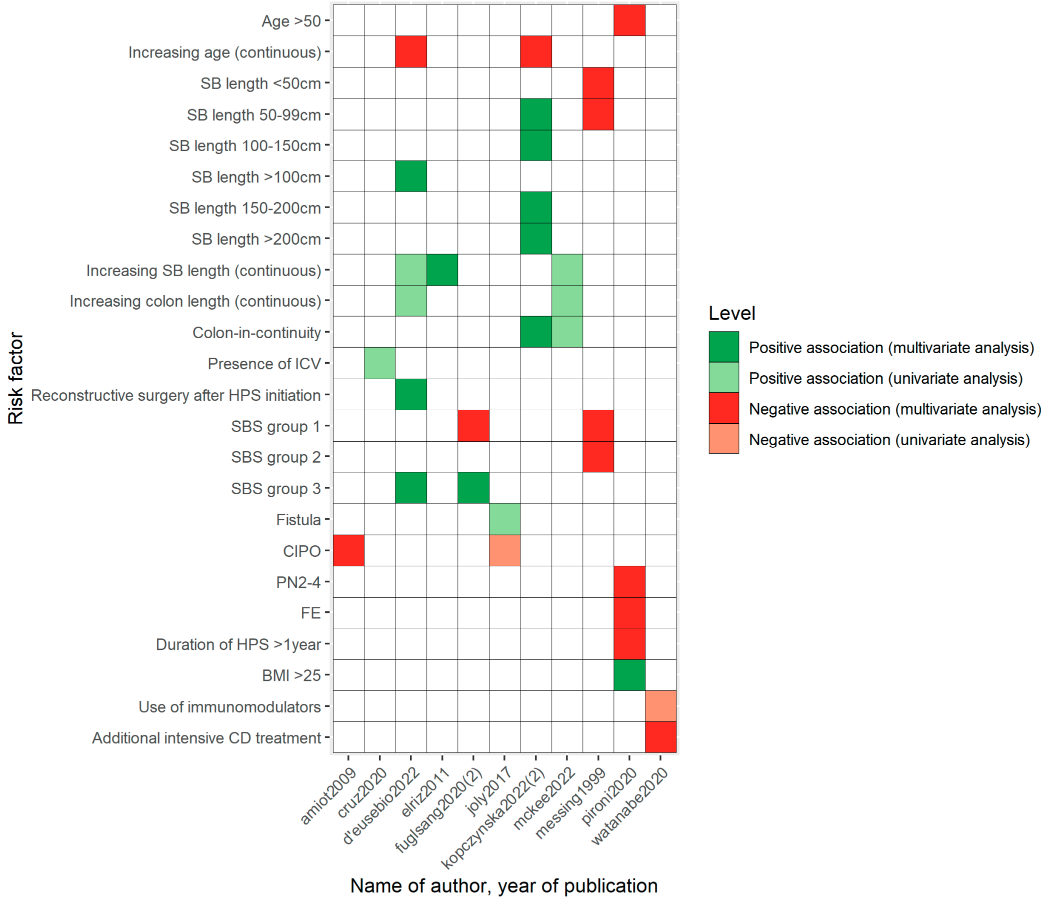

Supplementary Figure S7. Risk of Bias Chart for studies included in the systematic review.

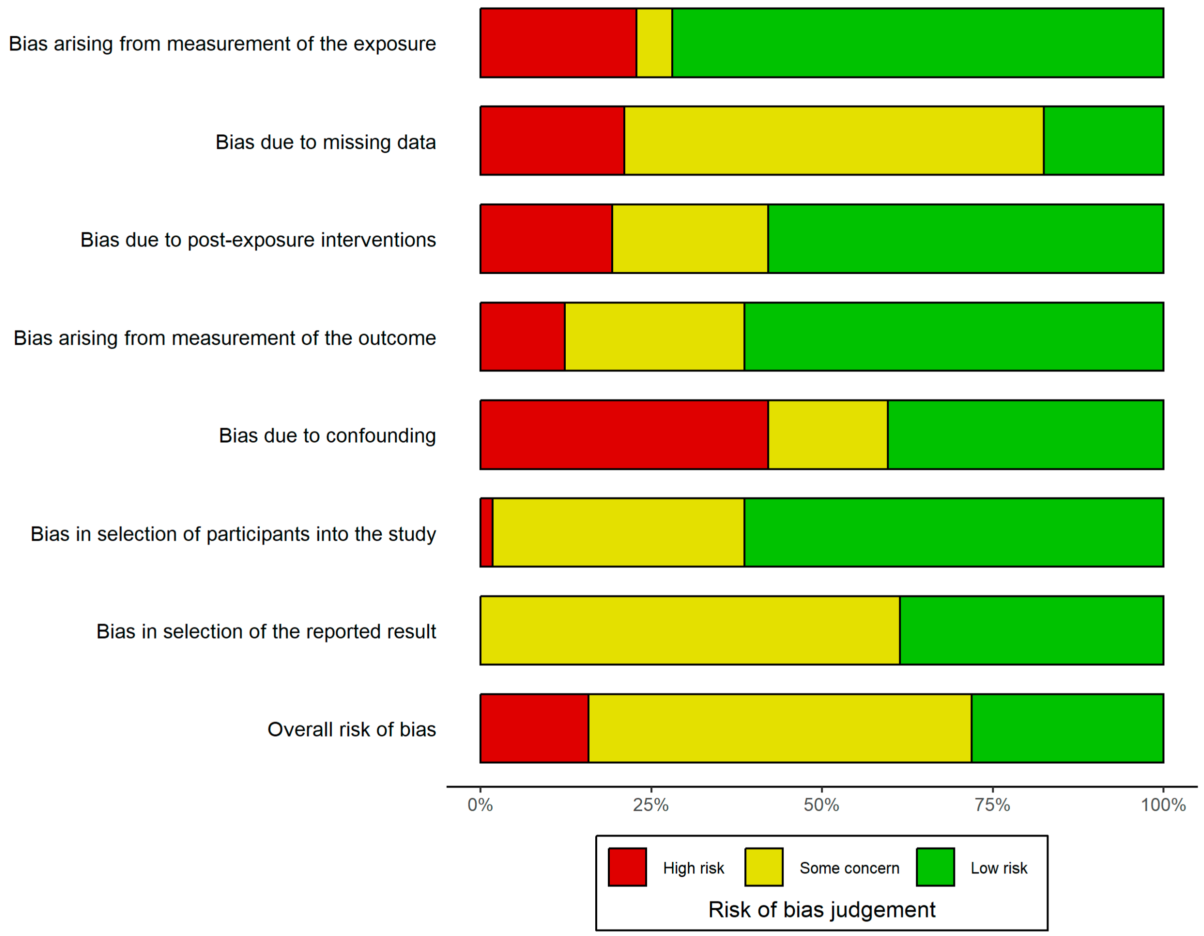

Supplement: Supplementary file 1 [file nutrients-18-02123-s001.zip › nutrients-4393845-supplementary.pdf]
